# Red Yeast Rice: A Systematic Review of the Traditional Uses, Chemistry, Pharmacology, and Quality Control of an Important Chinese Folk Medicine

**DOI:** 10.3389/fphar.2019.01449

**Published:** 2019-12-02

**Authors:** Bo Zhu, Fangyuan Qi, Jianjun Wu, Guoqing Yin, Jinwei Hua, Qiaoyan Zhang, Luping Qin

**Affiliations:** ^1^School of Pharmacy, Zhejiang Chinese Medical University, Hangzhou, China; ^2^Department of Pharmacy, Hangzhou Twin-Horse Biotechnology Co., Ltd., Hangzhou, China; ^3^Institute of Traditional Chinese Medicine, Lishui Academy of Agricultural and Forestry Sciences, Lishui, China

**Keywords:** red yeast rice, *Monascus purpureus*, traditional use, chemical constituent, biological activity, quality control

## Abstract

Red yeast rice (RYR), a Chinese traditional folk medicine produced by the fermentation of cooked rice kernels with a Monascaceae mold, *Monascus purpureus*, has long been used to treat blood circulation stasis, indigestion, diarrhea, and limb weakness in East Asian countries. This article provides a systematic review of the traditional uses, chemistry, biological activities, and toxicology of RYR to highlight its future prospects in the field of medicine. The literature reviewed for this article was obtained from the Web of Science, Elsevier, SciFinder, PubMed, CNKI, ScienceDirect, and Google Scholar, as well as Ph.D. and M.Sc. dissertations, published prior to July 2019. More than 101 chemical constituents have been isolated from RYR, mainly consisting of monacolins, pigments, organic acids, sterols, decalin derivatives, flavonoids, polysaccharides, and other compounds. Crude extracts of RYR, as well as its isolated compounds, possess broad pharmacological properties with hypolipidemic, anti-atherosclerotic, anti-cancer, neurocytoprotective, anti-osteoporotic, anti-fatigue, anti-diabetic, and anti-hypertensive activities. However, further studies are needed to characterize its diverse chemical constituents and the toxicological actions of the main bioactive compounds. New pharmacological trials addressing the overlooked traditional uses of RYR, such as in the treatment of indigestion and diarrhea, are required.

## Introduction

Red yeast rice (RYR) is a traditional Chinese medicine and food supplement popular in East Asian countries such as China, Japan, Korea, and Thailand (Patel, 2016). It is produced by the fermentation of cooked rice kernels with a Monascaceae mold, *Monascus purpureus*, which turns rice into reddish purple kernels due to its pigmentation capability (Kalaivani et al., 2010). As part of the Asian diet, RYR is used as a food additive to enhance the color of meat, fish, and soybean products. It is also recognized as a folk medicine for rejuvenating the body, promoting blood circulation, and restoring stomach balance (Mazzanti et al., 2017). With the increasing tendency of Western countries to use statins, a group of drugs used to treat hyperlipidemia, RYR has attracted considerable research attention (Burke, 2015). RYR has been reported to possess numerous biological properties with hypolipidemic, anti-atherosclerotic, anti-cancer, neurocytoprotective, hepatoprotective, anti-osteoporotic, anti-fatigue, anti-diabetic, anti-obesity, immunomodulatory, anti-inflammatory, anti-hypertensive, and anti-microbial activities. RYR can also improve the quality of eggs. Chemical analyses have revealed that RYR contains monacolins, pigments, organic acids, amino acids, sterols, decalin derivatives, flavonoids, lignans, coumarin, terpenoids, and polysaccharides. However, only a few of these compounds have been screened in bioactivity assays, and their structures have not been sufficiently characterized. Although RYR is effective in treating various infections, the safety of its chemical constituents has not been defined. In addition, the quality control of RYR has not been investigated, and there is a lack of pharmacological information on the traditional uses of RYR.

In this article, we provide an up-to-date and comprehensive literature analysis of RYR and address its traditional uses, chemistry, pharmacological activities, possible molecular mechanisms, and safety. A critical evaluation of pharmacological studies in terms of the ethnomedical use of RYR was also performed. This information may provide new insights on RYR or its active ingredients, and help seek effective intervention strategies for the prevention and treatment of diseases, design clinical trials of bioactive compounds in RYR in future research, and develop fungal-medicines as well as edible products containing these functional properties.

## Materials and Methods

Information on studies involving RYR was gathered *via* the Internet using Google Scholar, Baidu Scholar, Elsevier, Web of Science, PubMed, CNKI, ScienceDirect, SciFinder, and Scopus, and a library search was performed of articles published in classic books of Chinese Herbal Medicine, local herbal encyclopedias, and Ph.D. and M.Sc. dissertations. The key words used were RYR, red mold rice, *M. purpureus*, secondary metabolites, chemistry, biological activity, pharmacology, medicinal use, safety, quality control, toxicology, and other related words (Zhu et al., 2018). The MycoBank database (http://mycobank.org) was used to validate the scientific names of *M*. *purpureus*.

## Mycology

RYR (**Figures S1A, B**; also known as “*Hongqu*” or “*angkak*” in China, and ‘*red koji*’ in Japan) is a remedy belonging to Traditional Chinese Medicine (TCM). Nowadays, it is also used as a dietary supplement in Western countries (Hong et al., 2008b; Fung et al., 2012). It is produced by the fermentation of steamed rice with a food fungus of the *Monascus* genus, usually *M. purpureus* Went. Apart from *M. purpureus*, other related nonpathogenic molds of the *Monascus* genus, such as *M. ruber*, *M. anka*, and *M. pilosus*, are also used for RYR production in Japan, Korea, India, and Thailand (Cheng et al., 2013; Hong et al., 2017). In TCM, however, *M. purpureus* is the only accepted medicinal fungus for RYR fermentation (Chinese Pharmacopoeia, 2015). According to the MycoBank database (http://mycobank.org), *M. purpureus* (MB#235390) is the only accepted name for the fungus, and it has six synonyms, including *M. albidus* var. *albidus*, *M. anka* Nakaz. and K. Satô, *M. araneosus* K. Satô, *M. major* K. Satô, *M. rubiginosus* K. Satô, and *M. albidus* var. *glaber* K. Satô.

*M. purpureus*, which was largely described by Went in 1985, belongs to family Monascaceae, class Eurotiomycetes in Ascomycota (Huang et al., 2007). It can grow rapidly at a temperature of 25°C to 30°C on Potato Dextrose Agar or Luria Bertani mediums, and it favors conditions with a humidity of 65% to 85% (**Figures S1C, D**) (Il Gum et al., 2017). *M. purpureus* mostly breeds in asexual generation style, with many botryoid conidium. In general, stromata are solitary to gregarious, colony margins are short pulvinate to filiform, the ascocarp wall pectizes to membrane-like, ascospores are oval, the surface is smooth, and the color is red to nearly gray (**Figures S1E, F**) (Wei, 1979).

## Traditional Uses

RYR was first recorded in the Local Chronicles of Gutian (⟪古田县志⟫), which dates back to the Tang Dynasty (A.D. 618–907) (Lin, 2017). RYR has also been recorded in many ancient texts, such as Qing Yi Lu (⟪清异录⟫) (from the Five Dynasties and North Song Dynasty, A.D. 907–1127), Hai Lu Sui Shi (⟪海录碎事⟫) (Song Dynasty, A.D. 960–1279), and Tian Gong Kai Wu (⟪天工开物⟫) (Ming Dynasty, A.D. 1368–1644), and it is widely used in the Chinese culture as a food preservative, flavor enhancer, and food-coloring agent for fish sauces, rice wines, red soybean curd, pickled vegetables, and salted meats (Burke, 2015; Lin, 2017). In TCM, according to Materia Medica in Daily Use (⟪日用本草⟫) (Yuan Dynasty, A.D. 1271–1368) and the Compendium of Herbology (⟪本草纲目⟫) (A.D. 1552–1578), RYR is slightly mild and sweet, without significant toxicity, and it goes to the liver, spleen, and large intestines. It has been used to treat indigestion, diarrhea, blood circulation stasis, and limb weakness for more than 700 years (Chinese Pharmacopoeia, 2015; Patel, 2016). In some western countries, including the United States, Netherlands, and Italy, RYR has been used as an alternative to statin therapy, especially in patients who are intolerant to standardized therapy due to statin-associated myalgia or those who are opposed to taking statins (Childress et al., 2013).

RYR has been widely used in China as both food and medicine. RYR, as an ingredient, is found in more than 155 healthy foods, with purported curative powers ranging from reducing blood lipid levels and enhancing immunity to reducing blood pressure and alleviating fatigue (http://www.da.yaozh.com, last accessed on 28/07/2019). RYR-based products include *Hongqu Jiaonang*, *Yanjitangpai Kouyiling Ruanjiaonang*, and *Caizhiyuan Q* *_10_* *Ruanjiaonang*. Moreover, RYR has been used in at least 24 TCM preparations and at least 24 prescriptions for treating various chronic diseases (http://www.da.yaozh.com, last accessed on 28/07/2019). Commonly used Chinese herbal prescriptions containing RYR are listed in **Table S1**. “*Hong Qu Jiu*,” “*Huo Tui Hong Qu San*,” and “*Huang Lian Hong Qu Tang*” are Chinese prescriptions, whose use has been recorded in many ancient books, including the Compendium of Herbology (⟪本草纲目⟫), Following Traditional Customs Medical (⟪医学从众录⟫), and Gynecological Treatment of the Zhulin Temple (⟪竹林女科证治⟫). “*Xue Zhi Kang Pian*,” “*Xue Zhi Kang Jiao Nang*,” and “*Xin Huang Pian*” are folk medicines that have been accredited by the China State Food and Drug Administration. These entities are manufactured and sold in China to treat hyperlipemia, fatigue, and diarrhea (Chinese Pharmacopoeia, 2015).

## Chemistry

RYR is comprised of various chemical constituents, including monacolins (**1**–**23**), pigments (**24**–**48**), organic acids (**49**–**55**), amino acids (**56**, **57**), sterols (**58**–**66**), decalin derivatives (**67**–**73**), flavonoids (**74**, **75**), lignans (**76**, **77**), coumarin (**78**), terpenoids (**79**–**83**), and others (**84**–**92**). In addition, polysaccharides, such as EPS-1, EPS-2, EPS-3, EPS-4, EPS-5, MPS-1, MPS-2, MPS-3, and monascan, have been isolated from RYR (**Table 1**). According to previously published chemical studies, monacolins and pigments are the most abundant and bioactive constituents in RYR. Among these, monacolins [e.g. monacolin K (MK), also known as lovastatin], and pigments (e.g. monascin, rubropunctatin, and rubropunctamine) have been extensively investigated and considered to have potential benefits.

**Table 1:** Chemical constituents isolated from red yeast rice.

Class	Compound	Molecular formula	CAS registry number	Reference(s)
Monacolins	Monacolin K (lovastatin) **1**	C_24_H_36_O_5_	75330-75-5	(Ma et al., 2000; Liu et al., 2010; Wang, 2010; Ge et al., 2012; Maisarah et al., 2012; Zhu et al., 2012b; Tan, 2015; Zhang et al., 2016)
	Monacolin L **2**	C_19_H_28_O_3_	79394-47-1	(Ma et al., 2000; Zhu et al., 2012b; Xu et al., 2017)
	Monacolin Q **3**	C_19_H_22_O_2_	1879038-89-7	(Zhang et al., 2016)
	Monacolin R **4**	C_19_H_28_O_3_	1879038-90-0	(Zhang et al., 2016)
	Monacolin S **5**	C_24_H_38_O_7_	81693-02-9	(Zhang et al., 2016)
	Dehydromonacolin J **6**	C_19_H_26_O_3_	1355394-51-2	(Zhu et al., 2012b)
	Dehydromonacolin K **7**	C_24_H_34_O_4_	109273-98-5	(Ma et al., 2000; Zhu et al., 2012a; Zhang et al., 2016)
	Dehydromonacolin L **8**	C_19_H_26_O_2_	1355394-52-3	(Zhu et al., 2012a; Zhang et al., 2016)
	Dehydromonacolin N **9**	C_21_H_28_O_4_	1355394-50-1	(Zhu et al., 2012a)
	Dihydromonacolin K **10**	C_24_H_38_O_5_	77517-29-4	(Ma et al., 2000; Zhu et al., 2012a; Zhang et al., 2016)
	Dihydromonacolin L **11**	C_19_H_30_O_3_	86827-77-2	(Zhang et al., 2016)
	Dihydromonacolin-MV **12**	C_24_H_38_O_5_	935846-59-6	(Dhale et al., 2007a)
	Dehydromonacolin-MV2 **13**	C_19_H_26_O_5_	1018346-91-2	(Dhale et al., 2007b)
	Ethyl ester of monacolin K **14**	C_26_H_42_O_6_	77517-31-8	(Zhu et al., 2012b)
	Methyl ester of the hydroxyl acid form of monacolin K **15**	C_25_H_40_O_6_	77934-80-6	(Ma et al., 2000; Zhu et al., 2012b)
	Methyl ester of the hydroxy acid form of monacolin L **16**	C_20_H_32_O_4_	312710-94-4	(Ma et al., 2000)
	α,β-Dehydromonacolin S **17**	C_24_H_36_O_6_	1879038-91-1	(Zhang et al., 2016)
	α,β-Hydromonacolin Q **18**	C_19_H_24_O_3_	118045-32-2	(Zhang et al., 2016)
	3α-Hydroxy-3,5-dihydromonacolin L **19**	C_19_H_30_O_4_	119786-66-2	(Zhang et al., 2016)
	3β-Hydroxy-3,5-dihydromonacolin L **20**	C_19_H_30_O_4_	1879038-92-2	(Zhang et al., 2016)
	α,β-Dehydrodihydromonacolin K **21**	C_24_H_36_O_4_	312710-92-2	(Zhu et al., 2012b; Zhang et al., 2016)
	α,β-Dehydrodihydromonacolin L **22**	C_19_H_28_O_2_	531523-94-1	(Zhu et al., 2012b)
	(1S,2S,4aR,6S,8-S,8aS,3′S,5′R,2″S)-Methyl 1,2,4a,5,6,7,8,8a-octahydro-3′,5′-dihydroxy-2,6-dimethyl-8-[(2-methyl-1-oxobutyl)oxy]-1-naphthaleneheptanoate **23**	C_25_H_42_O_6_	101834-04-2	(Zhu et al., 2012b)
Pigments	Rubropunctamine **24**	C_21_H_23_NO_4_	13552-06-2	(Wild et al., 2002a; Wild et al., 2002b; Wild et al., 2003; Akihisa et al., 2005; Ferse et al., 2012)
	Rubropunctatin **25**	C_21_H_22_O_5_	13471-84-6	(Wild et al., 2002a; Wild et al., 2002b; Wild et al., 2003; Akihisa et al., 2005; Xu et al., 2017)
	Monascorubramine **26**	C_23_H_27_NO_4_	1003392-65-1	(Wild et al., 2002b; Wild et al., 2003; Akihisa et al., 2005; Ferse et al., 2012)
	Monascorubrin **27**	C_23_H_26_O_5_	13283-85-7	(Wild et al., 2002b; Wild et al., 2003; Akihisa et al., 2005)
	Monascin **28**	C_21_H_26_O_5_	3567-98-4	(Wild et al., 2002a; Wild et al., 2002b; Wild et al., 2003; Akihisa et al., 2005; Su et al., 2005; Cheng et al., 2010b; Ferse et al., 2012)
	Ankaflavin **29**	C_23_H_30_O_5_	50980-32-0	(Wild et al., 2002b; Wild et al., 2003; Akihisa et al., 2005; Su et al., 2005; Cheng et al., 2010b; Ferse et al., 2012)
	Xanthomonasin A **30**	C_21_H_24_O_7_	140375-37-7	(Wild et al., 2002b; Akihisa et al., 2005)
	Xanthomonasin B **31**	C_23_H_28_O_7_	146445-98-9	(Akihisa et al., 2005)
	Monankarin A **32**	C_20_H_22_O_6_	182161-52-0	(Hossain et al., 1996; Wild et al., 2002b)
	Monasfluore A **33**	C_21_H_24_O_5_	1004537-75-0	(Huang et al., 2008; Cheng et al., 2010a; Hsu et al., 2010; Ferse et al., 2012)
	Monasfluore B **34**	C_23_H_28_O_5_	1004537-76-1	(Huang et al., 2008; Cheng et al., 2010a; Hsu et al., 2010; Ferse et al., 2012)
	Monapurone A **35**	C_20_H_26_O_4_	1263777-47-4	(Li et al., 2010)
	Monapurone B **36**	C_21_H_28_O_4_	1263777-50-9	(Li et al., 2010)
	Monapurone C **37**	C_21_H_28_O_4_	1263777-48-5	(Li et al., 2010)
	Monascopyridine A **38**	C_21_H_25_NO_4_	604786-54-1	(Wild et al., 2003; Ferse et al., 2012)
	Monascopyridine B **39**	C_23_H_29_NO_4_	604786-55-2	(Wild et al., 2003; Ferse et al., 2012)
	Monascopyridine C **40**	C_20_H_27_NO_3_	909094-27-5	(Knecht et al., 2006; Hsu et al., 2010; Ferse et al., 2012)
	Monascopyridine D **41**	C_22_H_31_NO_3_	909094-28-6	(Knecht et al., 2006; Hsu et al., 2010; Ferse et al., 2012)
	Monascopyridine E **42**	C_21_H_27_NO_4_	1313735-11-3	(Ferse et al., 2012)
	Monascopyridine F **43**	C_23_H_31_NO_4_	1313735-13-5	(Ferse et al., 2012)
	Monapurfluore A **44**	C_23_H_32_O_4_	1259424-24-2	(Hsu et al., 2010)
	Monapurfluore B **45**	C_23_H_32_O_4_	1259424-22-0	(Hsu et al., 2010)
	4-[2,4-Dihydroxy-6-(3-hydroxybutanethioyloxy)-3-methylphenyl]-3,4-dihydroxy-3,6-dimethylheptanoic acid **46**	C_20_H_30_O_8_S	910788-93-1	(Campoy et al., 2006)
	9-Hexanoyl-3-(2-hydroxypropyl)-6a-methyl-9,9a-dihydro-6H-furo[2,3-h]isochromene-6,8(6aH)-dione **47**	C_21_H_26_O_6_	910788-92-0	(Campoy et al., 2006)
	Monapilosusazaphilone **48**	C_23_H_32_O_5_	1444202-34-9	(Cheng et al., 2013; Zhang et al., 2016)
Organic acids and amino acids	Linoleic acid **49**	C_18_H_32_O_2_	60-33-3	(Zhang et al., 2018a)
	α-Linolenic acid **50**	C_18_H_30_O_2_	463-40-1	(Zhang et al., 2018a)
	Citrinin **51**	C_13_H_14_O_5_	1086-03-9	(Wild et al., 2002a; Wild et al., 2002b; Wild et al., 2003)
	1-Heptadecanecarboxylic acid **52**	C_18_H_36_O_2_	57-11-4	(Tan, 2015)
	1-Pentadecanecarboxylic acid **53**	C_16_H_32_O_2_	57-10-3	(Tan, 2015)
	2-Hydroxyoctadecanoic acid **54**	C_18_H_36_O_3_	629-22-1	(Tan, 2015)
	5-(2′-Hydroxy-6′-methyl phenyl)-3-methylfuran-2-carboxylic acid **55**	C_13_H_12_O_4_	2060554-52-9	(Ji et al., 2018)
	(+)-Monascumic acid **56**	C_10_H_17_NO_4_	673477-38-8	(Akihisa et al., 2004; Akihisa et al., 2005)
	(−)-Monascumic acid **57**	C_10_H_17_NO_4_	673477-39-9	(Akihisa et al., 2004; Akihisa et al., 2005)
Sterols	Ergosterol **58**	C_28_H_44_O	57-87-4	(Cheng et al., 2010b; Wang, 2010; Ge et al., 2012; Tan, 2015)
	Stigmasterol **59**	C_29_H_48_O	83-48-7	(Wang, 2010; Ge et al., 2012; Tan, 2015)
	β-Sitosterol **60**	C_29_H_50_O	83-46-5	(Tan, 2015)
	3β-Hydroxylstigmast-5-en-7-one **61**	C_29_H_48_O_2_	2034-74-4	(Cheng et al., 2013)
	3β-Hydroxystigmasta-5,22-dien-7-one **62**	C_29_H_46_O_2_	36449-99-7	(Cheng et al., 2013)
	6β-Hydroxystigmast-4-en-3-one **63**	C_29_H_48_O_2_	36450-02-9	(Cheng et al., 2013)
	6β-Hydroxystigmasta-4,22-dien-3-one **64**	C_29_H_46_O_2_	36450-01-8	(Cheng et al., 2013)
	Daucosterol **65**	C_35_H_60_O_6_	474-58-8	(Tan, 2015)
	β-Sitosteryl palmitate **66**	C_44_H_76_O_2_	110716-42-2	(Cheng et al., 2010b)
Decalin derivatives	Monascusic acid A **67**	C_15_H_22_O_2_	1364517-38-3	(Zhang et al., 2009; Zhu et al., 2012a)
	Monascusic acid B **68**	C_15_H_22_O_2_	1364517-30-5	(Zhu et al., 2012a)
	Monascusic acid C **69**	C_15_H_22_O_2_	1364517-32-7	(Zhu et al., 2012a)
	Monascusic acid D **70**	C_15_H_20_O_2_	1364517-35-0	(Zhu et al., 2012a)
	Monascusic acid E **71**	C_15_H_20_O_2_	1364517-37-2	(Zhu et al., 2012a)
	Monascusic lactone A **72**	C_15_H_20_O_2_	1364517-28-1	(Zhu et al., 2012a)
	Heptaketide **73**	C_15_H_24_O_2_	531523-95-2	(Zhu et al., 2012a; Zhang et al., 2016)
Flavonoids, lignans, and coumarin	Daidzein **74**	C_15_H_10_O_4_	486-66-8	(Ji et al., 2018)
	Genistein **75**	C_15_H_10_O_5_	446-72-0	(Ji et al., 2018)
	5,5′-Dimethoxylariciresinol **76**	C_22_H_28_O_8_	116498-58-9	(Cheng et al., 2013)
	Lariciresinol **77**	C_20_H_24_O_6_	27003-73-2	(Cheng et al., 2013)
	Scopoletin **78**	C_10_H_8_O_4_	92-61-5	(Cheng et al., 2013)
Terpenoids	3-*epi*-Betulinic acid **79**	C_30_H_48_O_3_	472-15-1	(Cheng et al., 2010a)
	3-*epi*-Betulinic acid acetate **80**	C_32_H_50_O_4_	10376-50-8	(Cheng et al., 2010a)
	Friedelan-3-one **81**	C_30_H_50_O	559-74-0	(Cheng et al., 2010a)
	α-Cadinol **82**	C_15_H_26_O	481-34-5	(Cheng et al., 2010a)
	Anticopalol **83**	C_20_H_34_O	10395-43-4	(Cheng et al., 2010a)
Polysaccharides	EPS-1, EPS-2, EPS-3, EPS-4, EPS-5	—	—	(Jiang, 2016)
	MPS-1, MPS-2, MPS-3	—	—	(Jiang, 2016)
	Monascan	—	—	(Tian et al., 1998)
Other compounds	Peroxymonascuspyrone **84**	C_19_H_30_O_6_	2227383-92-6	(Cheng et al., 2010a)
	α-Tocospiro A **85**	C_29_H_50_O_4_	601490-40-8	(Cheng et al., 2010a)
	Spathulenol **86**	C_15_H_24_O	6750-60-3	(Kiem et al., 2005; Cheng et al., 2010a)
	Monascodilone **87**	C_15_H_12_O_4_	439668-12-9	(Wild et al., 2002a; Wild et al., 2002b)
	Monascustin **88**	C_10_H_18_N_2_O_3_	2083632-09-9	(Wei et al., 2017)
	N-*cis*-feruloylmethoxytyramine **89**	C_19_H_21_NO_5_	78510-19-7	(Cheng et al., 2013)
	Monaspurpurone **90**	C_13_H_14_O_5_	1262840-98-1	(Cheng et al., 2010b)
	*p*-Nitrophenol **91**	C_6_H_5_NO_3_	100-02-7	(Cheng et al., 2010b)
	1-Dotriacontanol **92**	C_32_H_66_	544-85-4	(Tan, 2015)

Dash (—) denotes no useful information found in the study.

### Monacolins

**Figure 1:**
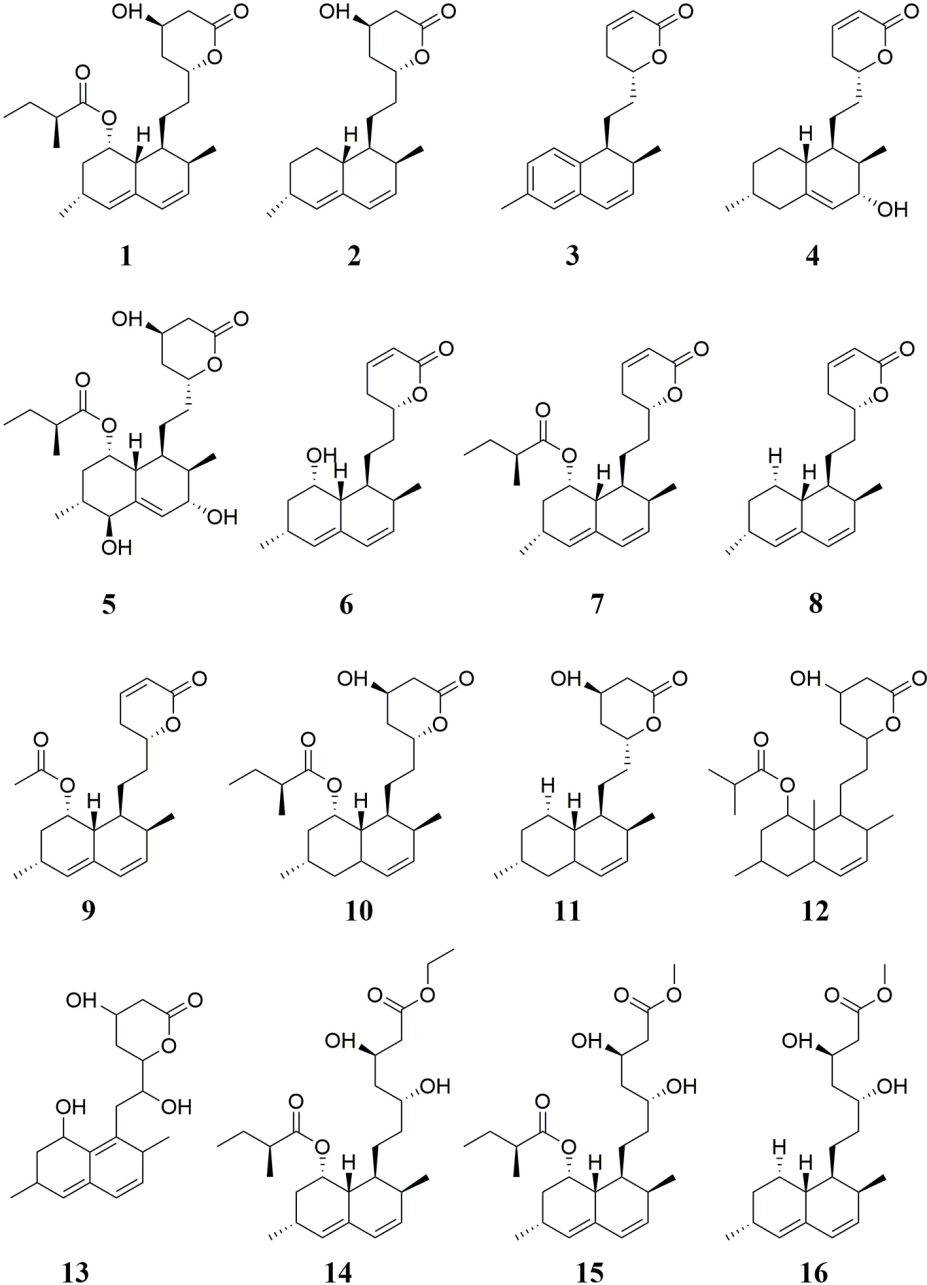
Monacolins isolated from RYR (I).

**Figure 2:**
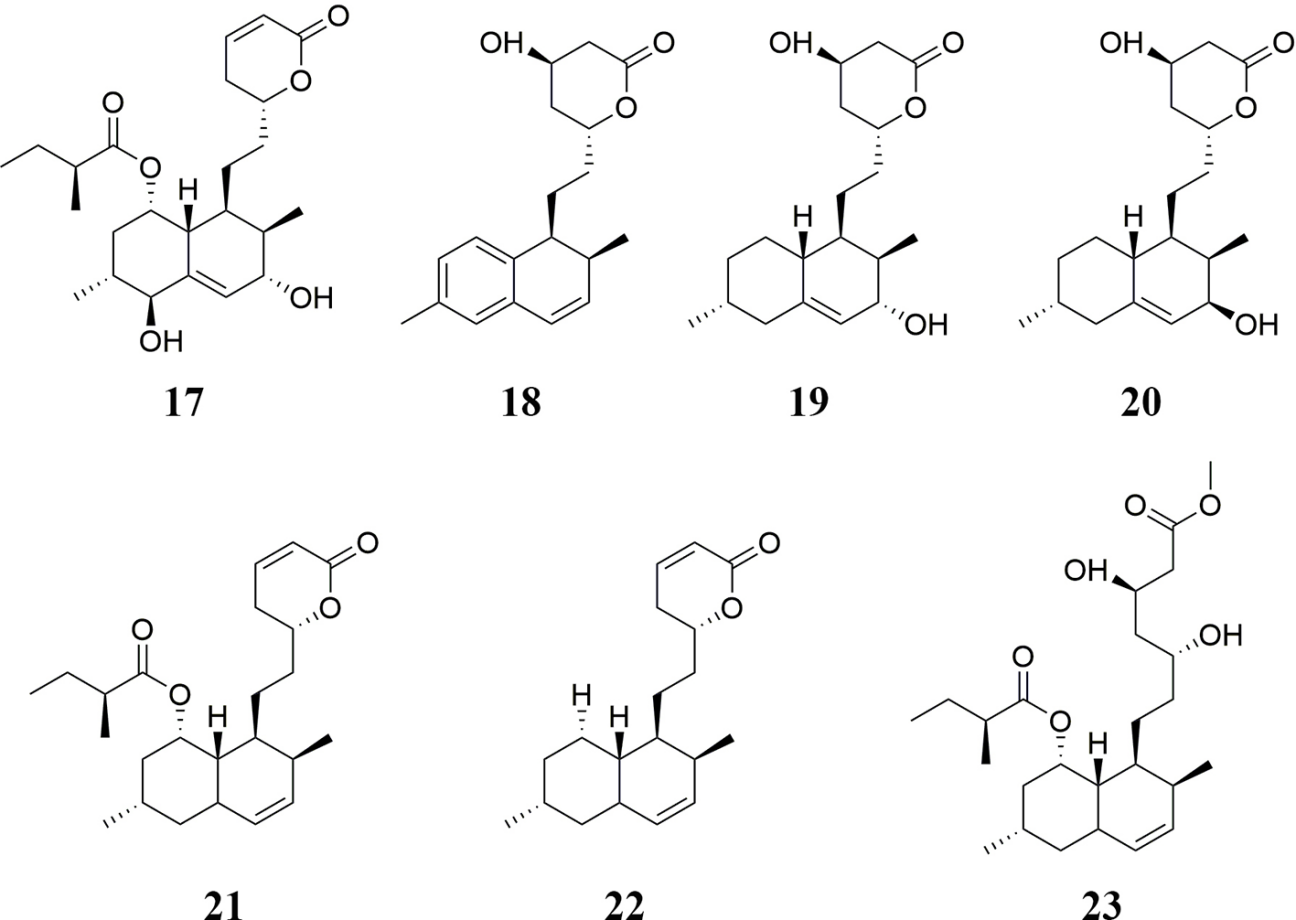
Monacolins isolated from RYR (II).

Monacolins are one of the main active ingredients in RYR. In total, twenty-three monacolins have been isolated from RYR, including MK **1**, monacolin L **2** (Ma et al., 2000), monacolin Q **3**, monacolin R **4**, monacolin S **5** (Zhang et al., 2016), mehydromonacolin J **6** (Zhu et al., 2012b), dehydromonacolin K **7**, dehydromonacolin L **8**, dehydromonacolin N **9**, dihydromonacolin K **10**, dihydromonacolin L **11** (Ma et al., 2000; Zhu et al., 2012a; Zhang et al., 2016), dihydromonacolin-MV **12**, dehydromonacolin-MV2 **13** (Dhale et al., 2007a; Dhale et al., 2007b), ethyl ester of MK **14**, methyl ester of the hydroxyl acid form of MK **15**, methyl ester of the hydroxy acid form of monacolin L **16** (Ma et al., 2000; Zhu et al., 2012b), α,β-dehydromonacolin S **17**, α,β-hydromonacolin Q **18**, 3α-hydroxy-3,5-dihydromonacolin L **19**, 3β-hydroxy-3,5-dihydromonacolin L **20**, α,β-dehydrodihydromonacolin K **21**, α,β-dehydrodihydromonacolin L **22**, and (1S,2S,4aR,6S,8-S,8aS,3′S,5′R,2″S)-methyl 1,2,4a,5,6,7,8,8a-octahydro-3′,5′-dihydroxy-2,6-dimethyl-8-[(2-methyl-1-oxobutyl)oxy]-1-naphthaleneheptanoate **23** (Zhu et al., 2012b; Zhang et al., 2016). Monacolins, especially RYR-derived MK, have been shown to have remarkable hypolipidemic effects (Chen, 2004) as well as anti-osteoporotic (Gutierrez et al., 2006), and anti-fatigue (Chen, 2004) activities. The chemical structures of these monacolins are shown in **Figures 1** and **2**.

### Pigments

**Figure 3:**
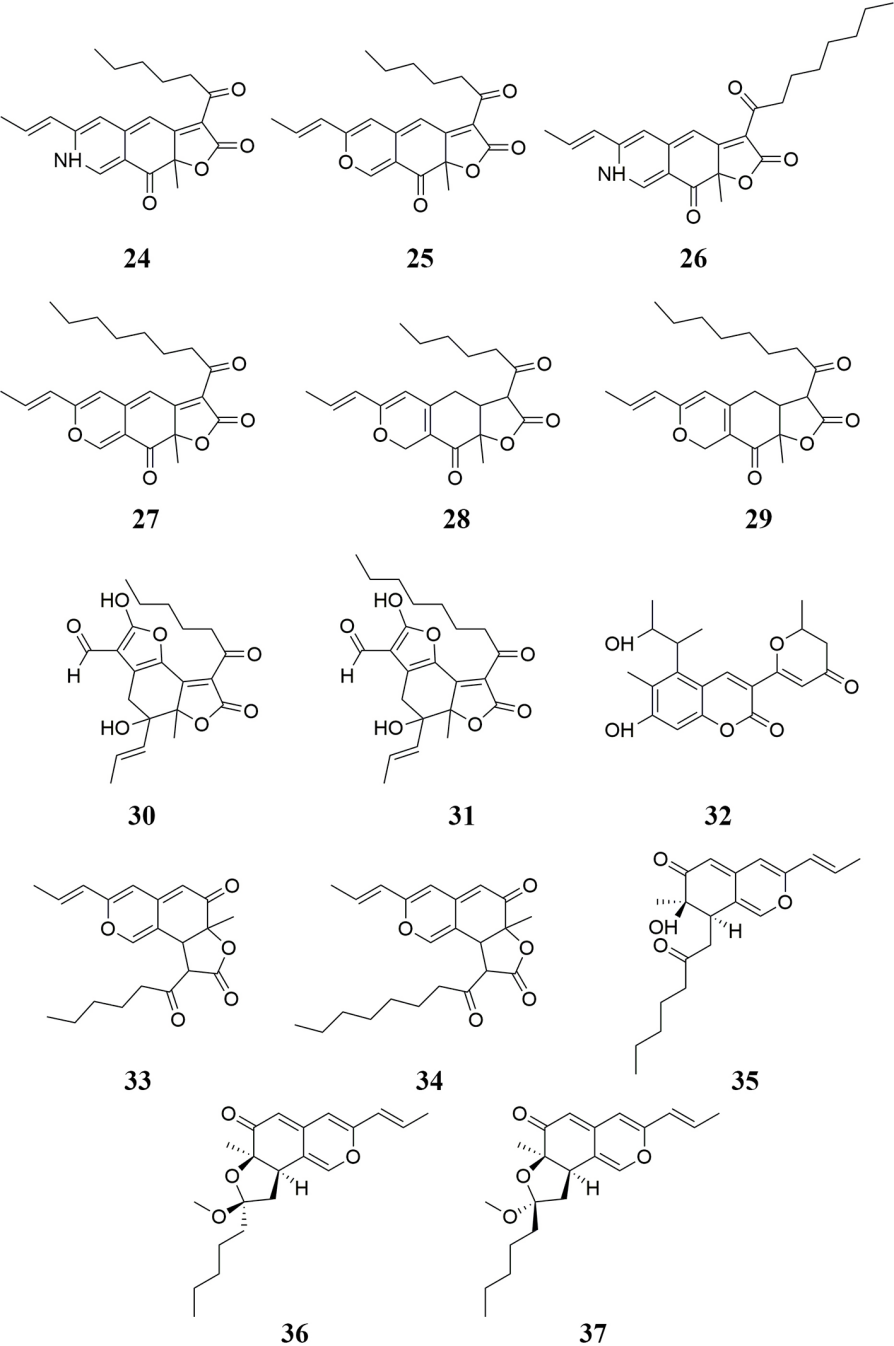
Pigments isolated from RYR (I).

**Figure 4:**
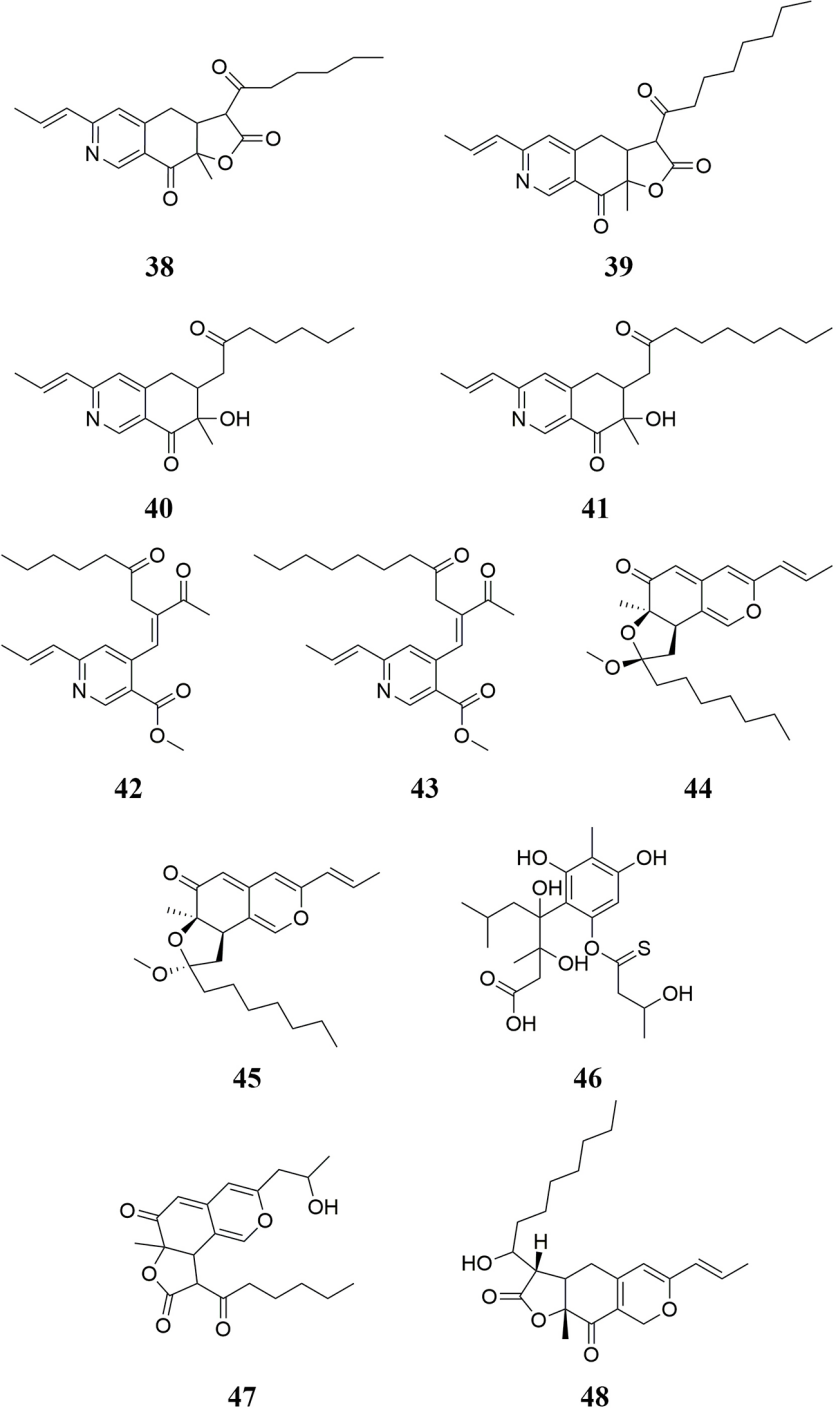
Pigments isolated from RYR (II).

Pigments are also important active compounds found in RYR. Twenty-five pigments have been isolated from RYR, including rubropunctamine **24**, rubropunctatin **25**, monascorubramine **26**, monascorubrin **27**, monascin **28**, ankaflavin **29**, xanthomonasin A **30**, xanthomonasin B **31**, monankarin A **32** (Wild et al., 2002b; Akihisa et al., 2005), monasfluore A **33**, monasfluore B **34** (Huang et al., 2008), monapurone A **35**, monapurone B **36**, monapurone C **37** (Li et al., 2010), monascopyridine A **38**, monascopyridine B **39**, monascopyridine C **40**, monascopyridine D **41**, monascopyridine E **42**, monascopyridine F **43** (Ferse et al., 2012), monapurfluore A **44**, monapurfluore B **45** (Hsu et al., 2010), 4-[2,4-dihydroxy-6-(3-hydroxybutanethioyloxy)- 3-methylphenyl]-3,4-dihydroxy-3,6-dimethylheptanoic acid **46**, 9-hexanoyl-3-(2-hydroxypropyl)-6a-methyl-9,9a-dihydro-6H-furo[2,3-h]isochromene-6,8(6aH)-dione **47** (Campoy et al., 2006), and monapilosusazaphilone **48** (Cheng et al., 2013). RYR-derived pigments possess hypolipidemic effects (Zhou et al., 2019) as well as anti-cancer (Xu et al., 2017), anti-fatigue (Chen, 2004), and anti-inflammatory (Hsu et al., 2010) activities. In addition, RYR pigments can be used to color yogurt red (Chen et al., 2012b). The chemical structures of these molecules are shown in **Figures 3** and **4**.

### Organic Acids and Amino Acids

Seven organic acids, including linoleic acid **49**, α-linolenic acid **50** (Zhang et al., 2018a), citrinin **51** (Wild et al., 2002b), 1-heptadecanecarboxylic acid **52**, 1-pentadecanecarboxylic acid **53**, 2-hydroxyoctadecanoic acid **54** (Tan, 2015), and 5-(2′-hydroxy-6′-methyl phenyl)-3-methylfuran-2-carboxylic acid **55** (Ji et al., 2018), as well as (+)-monascumic acid **56** and (−)-monascumic acid **57**, two amino acids (Akihisa et al., 2004; Akihisa et al., 2005), have been isolated from RYR. However, RYR-derived citrinin has been reported to have lethal effects on the kidney, and it may also act as a teratogen (harmful to the embryo or fetus) and genotoxin at high concentrations in cultured human lymphocytes (Patel, 2016). In addition, the two amino acids have been shown to have potent inhibitory effects on Epstein-Barr virus early antigen (EBV-EA) activation (Akihisa et al., 2005).

### Sterols

Nine sterols (ergosterol **58**, stigmasterol **59**, β-sitosterol **60**, 3β-hydroxylstigmast-5-en-7-one **61**, 3β-hydroxystigmasta-5,22-dien-7-one **62**, 6β-hydroxystigmast-4-en-3-one **63**, 6β-hydroxystigmasta-4,22-dien-3-one **64**, daucosterol **65**, and β-sitosteryl palmitate **66**) were isolated from RYR (Cheng et al., 2010b; Wang, 2010; Cheng et al., 2013; Tan, 2015). Among these, stigmasterol has been reported to possess hypolipidemic effects (Wang, 2010).

### Decalin Derivatives

**Figure 5:**
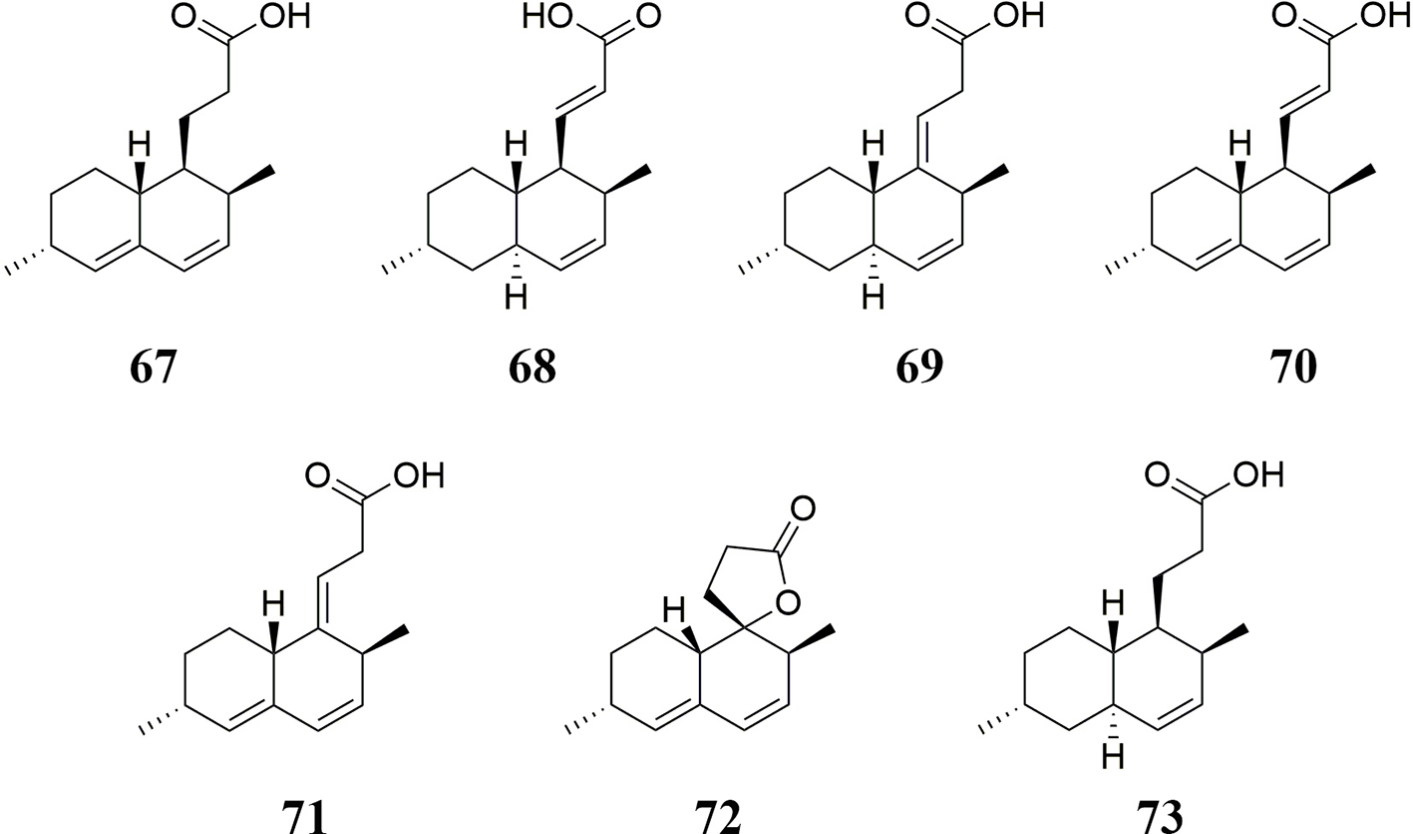
Decalin derivatives isolated from RYR.

Seven decalin derivatives, including monascusic acid A **67**, monascusic acid B **68**, monascusic acid C **69**, monascusic acid D **70**, monascusic acid E **71**, monascusic lactone A **72**, and heptaketide **73** were isolated from RYR (Zhang et al., 2009; Zhu et al., 2012a; Zhang et al., 2016). These decalin derivatives could suppress human T cell proliferation in a dose-dependent manner from 10 µmol/L to 100 µmol/L (Zhu et al., 2012a). The chemical structures of these decalin derivatives are shown in **Figure 5**.

### Flavonoids, Lignans, Coumarin, and Terpenoids

Two flavonoids, including daidzein **74** and genistein **75** (Ji et al., 2018), two lignans, including 5,5′-dimethoxylariciresinol **76** and lariciresinol **77**, and one coumarin (scopoletin **78**) (Cheng et al., 2013), as well as five terpenoids, including 3-*epi*-betulinic acid **79**, 3-*epi*-betulinic acid acetate **80**, Friedelan-3-one **81**, α-cadinol **82**, and anticopalol **83** (Cheng et al., 2010a), have been isolated from RYR. Presently, there are few studies investigating the pharmacological activities of these RYR-derived compounds.

### Polysaccharides

Nine polysaccharides, including EPS-1, EPS-2, EPS-3, EPS-4, EPS-5, MPS-1, MPS-2, MPS-3, and monascan, were isolated from RYR (Tian et al., 1998; Jiang, 2016). These polysaccharides are all composed of mannose, glucose, and galactose. EPS-1, EPS-2, EPS-3, EPS-4, and EPS-5 are composed of mannose, glucose, and galactose at a molar ratio of 0.364:0.415:0.221, whereas MPS-1, MPS-2, and MPS-3 are composed of mannose, glucose, and galactose at a molar ratio of 0.500:0.318:0.192 (Jiang, 2016). Monacan, a homogeneous polysaccharide with a molecular weight of ∼400,000 Da, is composed of mannose, glucose, and galactose at a molar ratio of 1:2:4, and its backbone is comprised of 1→3, 1→2, and 1→6 glucosidic bonds (Tian et al., 1998). Moreover, RYR-derived polysaccharides have been reported to possess anti-cancer (Ding, 2007) and immunomodulatory (Zhang et al., 2008) activities.

### Other Constituents

**Figure 6:**
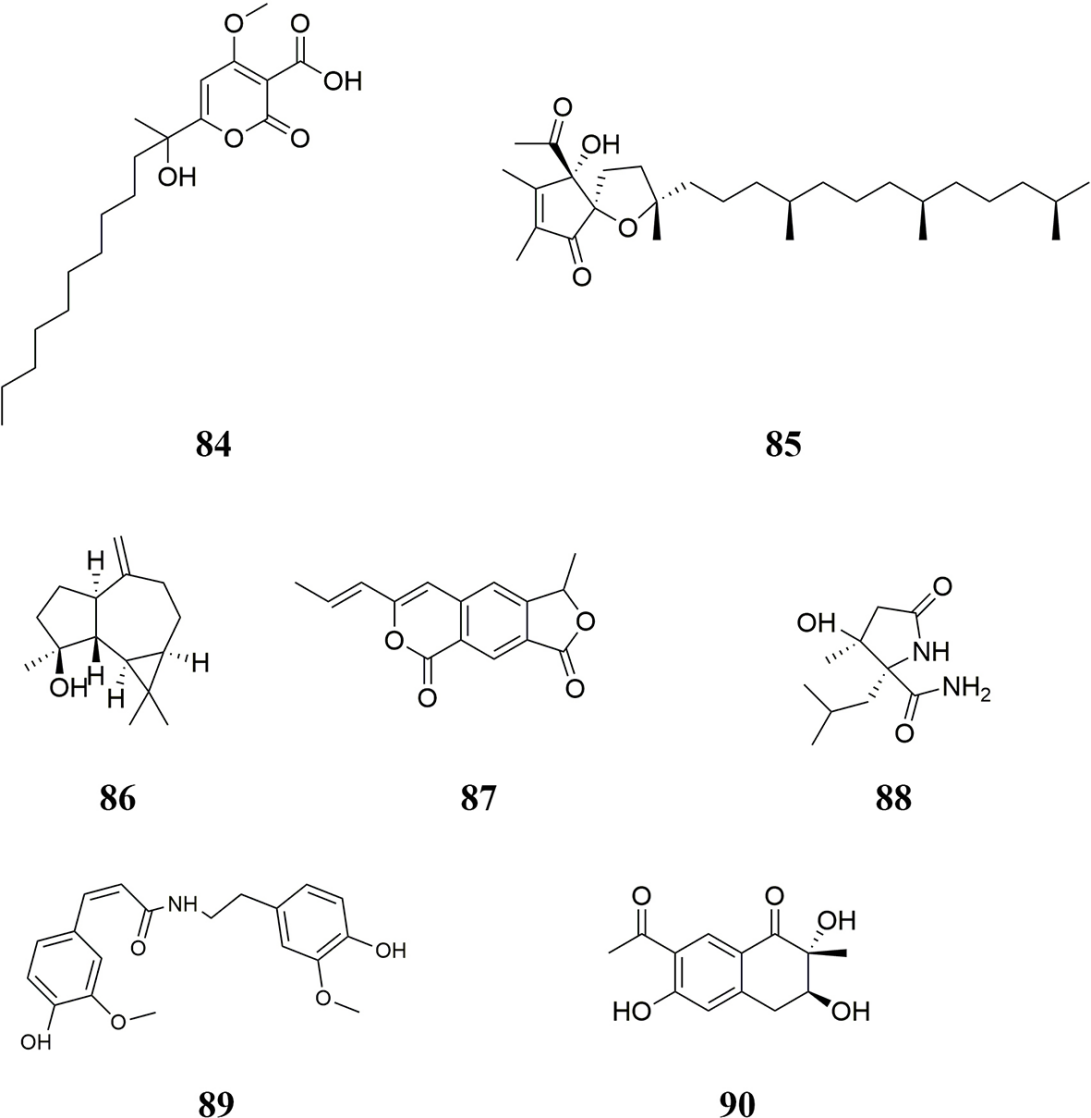
Other constituents isolated from RYR.

Nine other compounds, including peroxymonascuspyrone **84**, α-tocospiro A **85**, spathulenol **86** (Cheng et al., 2010a), monascodilone **87** (Wild et al., 2002a; Wild et al., 2002b), monascustin **88** (Wei et al., 2017), N-*cis*-feruloylmethoxytyramine **89** (Cheng et al., 2013), monaspurpurone **90**, *p*-nitrophenol **91** (Cheng et al., 2010b), and 1-dotriacontanol **92** (Tan, 2015), were isolated from RYR. The chemical structures of these compounds are shown in **Figure 6**.

## Pharmacological Activities

**Figure 7:**
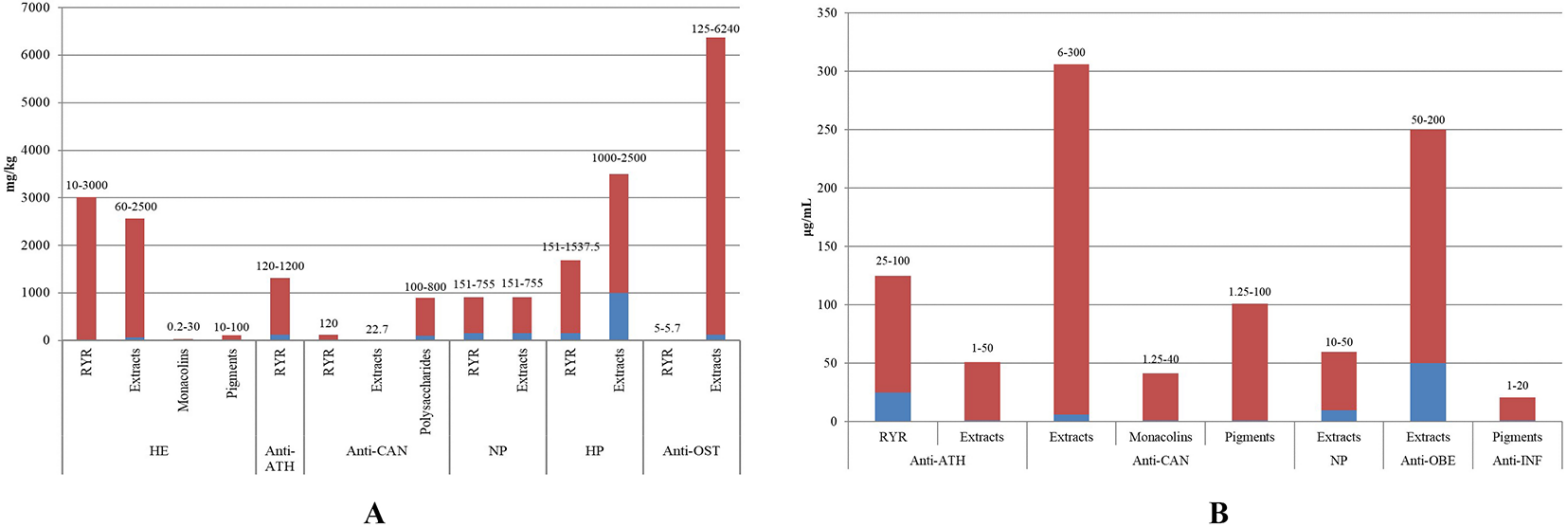
Dose-related effects of RYR extracts against various disorders *in vivo* **(A)** and *in vitro* **(B)** (HE, hypolipidemic effect; anti-ATH, anti-atherosclerotic activity; anti-CAN, anti-cancer activity; NP, neurocytoprotective activity; HP, hepatoprotective effect; anti-OST, anti-osteoporotic activity; anti-OBE, anti-obesity activity; anti-INF, anti-inflammatory activity.

RYR has been the subject of several pharmacological investigations due to its various ethnomedicinal uses. Studies have demonstrated that RYR exhibits a wide range of biological properties with hypolipidemic, anti-atherosclerotic, anti-cancer, neurocytoprotective, hepatoprotective, anti-osteoporotic, anti-fatigue, anti-diabetic, anti-obesity, immunomodulatory, anti-inflammatory, and anti-hypertensive activities. Among these, hypolipidemic and anti-atherosclerotic activities are most pronounced given that RYR is thought to play curative roles in resolving turbidity, invigorating blood circulation, and resolving blood stasis. These effects are summarized in **Table 2** and discussed in greater detail in the following sections. The relationships between the doses of RYR and its medicinal properties are shown in **Figure 7**.

**Table 2:** Pharmacological activities of red yeast rice.

**Pharmacological activity**	**Tested substance**	**Model**	**Tested living system/organ/cell**	**Result**	**Dose range**	**Time period of application**	**Reference(s)**
Hypolipidemic effect	Monacolin K	High fat diet fed mice	Liver and serum	Improved serum HDL-C level, decreased serum TC, TG, LDL-C levels, and up-regulated liver LPL mRNA and LDLR mRNA expression	5–30 mg/kg	6 weeks	(Chen, 2004)
	Pigments	High fat diet fed mice	Liver and serum	Improved serum HDL-C level, decreased serum TG, LDL-C levels, and up-regulated liver LPL mRNA and LDLR mRNA expression	10–100 mg/kg	6 weeks	(Chen, 2004)
	Pigments	High fat diet fed mice	Liver, blood, epididymal adipose, and fresh fecal samples	Ameliorated serum lipid levels, suppressed hepatic lipid accumulation and steatosis, and modulated the relative abundance of functionally related microbial phylotypes	20 mg/kg	8 weeks	(Zhou et al., 2019)
	Red yeast rice capsule	Healthy people with hyperlipidemia	Blood	Reduced TC, LDL-C, and total triacylglycerol concentrations	48 mg/kg	8 weeks	(Heber et al., 1999)
	Red yeast rice capsule	Atherogenic diet fed rabbit	Blood and aortas	Reduced serum TG	0.4–1.35 g/kg	200 days	(Wei et al., 2003)
	Aqueous extracts	High fat diet fed mice	Blood	Alleviated blood lipid parameters	1–2.5 g/kg	8–12 weeks	(Lee et al., 2015)
	Red yeast rice powder	Hamsters	Blood and feces	Reduced plasma TC and triacylglycerol, increased excretion of fecal acidic sterols	1–3 g/kg	6 weeks	(Ma et al., 2009)
	Red yeast rice capsule	Dyslipidemia patients	Blood	Decreased TC and LDL-C levels	36 mg/kg	24 weeks	(Becker et al., 2009)
	Red yeast rice capsule	Patients screened	Blood	Decreased TC and LDL-C levels	—	16 weeks	(Bogsrud et al., 2010)
	Red yeast rice capsule	Dyslipidemia patients induced by second-generation antipsychotics (SGAs)	Blood	Decreased LDL-C level	4 mg/kg	30 days	(Bruno et al., 2018)
	Red yeast rice capsule	High cholesterol diet fed mice	Blood	Down-regulated TG, TC, and LDL−C levels, and up-regulated HDL-C level	300 mg/kg	6 weeks	(Ding et al., 2014)
	Red yeast rice capsule	Lean and healthy people	Blood	Reduced TC, LDL-C, Hs-CRP, and PWV levels	12 mg/kg	4 weeks	(Lithander and Mahmud, 2015)
	Monacolins	Healthy, mildly hypercholesterolemic people	Plasma	Reduced TC, LDL-C, non–high density lipoprotein cholesterol, matrix metalloproteinase 2/9 levels	0.2 mg/kg	4 weeks	(Cicero et al., 2013)
	Red rice yeast capsule	Hyperlipidemia patients	Blood	Increased adiponectin and lowered LDL-C and TC levels	12 mg/kg	8 weeks	(Lee et al., 2013b)
Anti-atherosclerotic activity	Aqueous and ethanol extracts	Bone marrow-derived proangiogenic cells (PACs)	PACs	Inhibited β-galactosidase activation, reduced oxidative stress, and improved heme oxygenase-1 level	12.5–50 μg/mL	12–48 h	(Liu et al., 2017)
	Aqueous and ethanol extracts	Tumor necrosis factor (TNF)-α-treated human aortic smooth muscle cells	HASMCs	Reduced TNF-α-stimulated MMP-2 and MMP-9 expression, down-regulated NF-κB activation and intracellular ROS formation	1–160 μg/mL	24 h	(Lin et al., 2011)
	Aqueous and ethanol extracts	Homocysteine treated human aortic endothelial cells	HAECs	Reduced HCY-stimulated endothelial adhesiveness, and down-regulated intracellular ROS formation	1–50 μg/mL	24 h	(Lin et al., 2008)
	Red yeast rice (XZK) powder	C57BL/6 mice	Mice and carotid arteries	Inhibited vulnerable plaque progression, decreased plaque area, and suppressed lesional endoplasmic reticulum stress	600–1200 mg/kg	8 weeks	(Shen et al., 2017)
	Red yeast rice	High fat diet fed mice	Heart, aortas, and plasma	Reduced plaque size, stabilized plaque, protected endothelium, and decreased number of lipid droplets and cholesterol calculi, and the levels of Hs-CRP, IL-6, and TNF-α	120 mg/kg	36 weeks	(Wu et al., 2017)
	Red yeast rice (XZK) powder	RAW264.7 cells	RAW264.7 cells	Reduced nicotinamide adenine dinucleotide phosphate oxidase activity, decreased membrane translocation of p47^phox^, and inhibited extracellular signal-regulated kinase 1/2 activity	25–100 μg/mL	2 h	(Li et al., 2011)
Anti-cancer activity	Red yeast rice capsule	Breast cancer patients	Breast cancer patients and their serum	Improved living quality, and decreased CA125, CA153, VEGF, and VEGFR2 levels	120 mg/kg	12 weeks	(Zhong, 2017)
	Aqueous extracts	HUVEC cells	HUVEC cells	Inhibited cells proliferation and migration, and decreased VEGFR2 mRNA expression level	10–50 μg/mL	24 h	(Zhong, 2017)
	Ethanol, and ethyl acetate extracts	MCF-7 human breast cancer cells	MCF-7 cells	Exhibited direct cytotoxic and proapoptotic effects	50–150 μg/mL	24–48 h	(Lee et al., 2013a)
	Methanol extracts	Caco-2 human colorectal adenocarcinoma cells	Caco-2 cells	Exhibited antiproliferative effect, deregulated some proteins expression	1–100 μmol/L (MK)	24 h	(Lin et al., 2007)
	Polysaccharides	Replanted S_180_ tumor mice	Mice and their tumor, spleen	Inhibited tumor growth, and improved body weight	800 mg/kg	2 weeks	(Ding, 2007)
	Rubropunctatin	K-562, SK-OV-3, and SNU-1 cells	K-562, SK-OV-3, and SNU-1 cells	Exhibited telomerase inhibitory effects by down-regulating gene expression of telomerase-related protein hTERT	1.25–40 μg/mL	48 h	(Xu et al., 2017)
	Monacolin L	K-562, SK-OV-3, and SNU-1 cells	K-562, SK-OV-3, and SNU-1 cells	Inhibited cancer cells proliferation and induced apoptosis	1.25–40 μg/mL	48 h	(Xu et al., 2017)
	Methylene chloride extracts	LNCaP human PCa cells	LNCaP human PCa cells	Inhibited cancer cell growth	6–150 μg/mL	48–72 h	(Hong et al., 2008a)
	Red yeast rice powder	SCID tumor mice induced by human prostate cancer cells	Tumor and serum	Reduced tumor volumes, decreased serum PSA levels, and gene expression of androgen synthesizing enzymes (HSD3B2, AKR1C3, and SRD5A1)	—	—	(Hong et al., 2011)
	Methylene chloride extracts	HCT-116 and HT-29 human colon cancer cells	HCT-116 and HT-29 cells	Inhibited both tumor cell growths and enhanced apoptosis	20–300 μg/mL	24–72 h	(Hong et al., 2008b)
	Ankaflavin	HepG2 and A549 cells	HepG2 and A549 cells	Stimulated apoptosis	15–30 μg/mL	48 h	(Su et al., 2005)
	Monapurfluores A and B, monascopyridines C and D	HepG2 and WiDr cell lines	HepG2 and WiDr cell lines	Inhibited cell growth	6.26–100 μg/mL	72 h	(Hsu et al., 2010)
Neurocytoprotective activity	Ethanol extracts	Neuronally differentiated PC12 cells	PC12 cells	Provided stronger neuroprotection, and repressed inflammatory response and oxidative stress.	10–25 μg/mL	48 h	(Lee et al., 2007b)
	Ethanol extracts	SD rats induced by Amyloid β, water maze, and passive avoidance tasks	Rats, cerebral cortex, and hippocampus	Reversed memory deficit, prevented amyloid β fibrils from being formed and deposited in hippocampus and further decrease Aβ40 accumulation	151–755 mg/kg	4 weeks	(Lee et al., 2007b)
	Red yeast rice power	Zn-deficient diet fed rats, water maze, and passive avoidance tasks	Rats, cortex, hippocampus, and blood	Improved antioxidant enzyme activities and neural activity to maintain cortex and hippocampus functions	151–755 mg/kg	4 weeks	(Lee et al., 2009)
	Lovastatin derivatives	PC12 cells induced by 6-OHDA	PC12 cells	Reduced apoptosis, caspase-3, -8, and -9 activities and intracellular calcium concentrations, stabilized mitochondrial membrane potential	12.5–100 μmol/L	24 h	(Lin et al., 2015)
	Ethanol extracts	Human neuroblastoma IMR32 cells induced by cholesterol	IMR32 cells	Suppressed cholesterol raised β-secretase activity, increased sAPPR secretion	10–50 μg/mL	48 h	(Lee et al., 2010)
	Red yeast rice power	Rats, water maze and passive avoidance tasks.	Rats, blood, and brain	Reversed the memory deficit, decreased thiobarbituric acid reactive substances and reactive oxygen species	151–755 mg/kg	4 weeks	(Lee et al., 2010)
Hepatoprotective effect	Red yeast rice power	Chronic alcohol-induced mice	Liver, kidney, and blood	Attenuated the increased serum transaminases levels, hepatic triglyceride and TC accumulation, elevated hepatic antioxidant ability, reduced hepatic cell damage (steatosis), and decreased tissue inflammatory cytokine levels	307.5–1,537.5 mg/kg	5 weeks	(Cheng and Pan, 2011)
	Red yeast rice power	High cholesterol diet fed mice	Liver and serum	Reduced insulin resistance, inhibited TNF-α expression, and increased PPARα mRNA and proteins expression levels	0.17–1 g/kg	8 weeks	(Luo, 2009)
	Red yeast rice (XZK) capsule	High fat diet fed mice	Liver and blood	Ameliorated dyslipidemia and fat accumulation in the liver; improved insulin resistance and ameliorated oxidative stress; lessened hepatic steatosis, necro-inflammation, and collagen deposition; and inhibited hepatic expression of TNF-α	300 mg/kg	6 weeks	(Hong et al., 2007)
	Red yeast rice power	Zn-deficient rats	Liver and blood	Inhibited serum ALT levels, increased hepatic antioxidase activity, suppressed the productions of ROS and proinflammatory cytokines	151–755 mg/kg	4 weeks	(Lee et al., 2012)
Anti-osteoporotic activity	Red yeast rice power	Bone defects rabbits	Rabbits	Promoted new bone formation	5.0–5.7 mg/kg	—	(Wong and Rabie, 2008)
	Red yeast rice extracts	UMR 106 cells	UMR 106 cells	Increased the optical density in the MTT assay and ALP activity	1–10 mg/mL	24–72 h	(Wong and Rabie, 2008)
	Methanol extracts	Osteoblast-like MC3T3-E1 cells	MC3T3-E1 cells	Stimulated cell proliferation and ALP activity	0.001–1.0 mg/mL	24 h, 5–25 days	(Cho et al., 2010)
	Ethanol extracts	Ovariectomy-induced bone loss rats	Femora and serum	Increased the bone mineral density, decreased the levels of bone turnover markers, including osteocalcin and tartrate resistant acid phosphatase activity	1.56–6.24 g/kg	20 weeks	(Wang et al., 2015)
	Ethanol extracts	Osteoblast cells	Osteoblast cells	Improved the osteoblast viabilities, enhanced the expression of Bmp2 and Bmp4 both at the mRNA and protein levels	—	48 h	(Wang et al., 2015)
	Methanol extracts	SD rats	Bones	Stimulated new bone formation	125–500 mg/kg	5 days or 2 days/week for 5 weeks	(Gutierrez et al., 2006)
Anti-fatigue activity	Monacolin K	Mice and swimming test	Liver and serum	Improved hepatic glycogen level, decreased serum urea nitrogen level, and increased swimming time	10–90 mg/kg	30 days	(Chen, 2004)
	Pigments	Mice and swimming test	Liver and serum	Improved hepatic glycogen level, decreased serum urea nitrogen level, and increased swimming time	50–200 mg/kg	30 days	(Chen, 2004)
	Aqueous extracts	Mice and swimming test	Mice	Increased swimming time	100 mg/kg	30 days	(Chen, 2004)
	Ethanol extracts	Mice and swimming test	Liver and serum	Improved hepatic glycogen level, decreased serum urea nitrogen level, and increased swimming time	100 mg/kg	30 days	(Chen, 2004)
	Red yeast rice pills	Dyslipidemia patients	Psychological and physical questionnaires	Induced less muscle fatigue and remained physical activity	24 mg/kg	4 weeks	(Xue et al., 2017)
	Red yeast rice power	Wistar rats and swimming endurance test	Rats and blood	Exhibited higher exercise time and blood glucose concentration, lowered blood lactate, blood urea nitrogen, and hemoglobin	1–5 g/kg	4 weeks	(Wang et al., 2006)
Anti-diabetic activity	Red yeast rice powder	Rats	Blood	Raised the release of ACh from nerve terminals, stimulated muscarinic M3 receptors, augmented the insulin release, and lowered plasma glucose	50–150 mg/kg	90 min	(Chen and Liu, 2006)
	Red yeast rice	Diabetic rats induced by streptozotocin	Rats, liver, and blood	Lowered plasma glucose, mRNA levels of phosphoenolpyruvate carboxykinase in liver, and reversed hyperphagia	50–350 mg/kg	2 weeks	(Chang et al., 2006)
	Red yeast rice (XZK)	Diabetic mice	Blood, islet, and pancreas	Decreased blood glucose level, improved glucose tolerance and insulin secretion, protected islets	300 mg/kg	8 weeks	(Wang et al., 2014)
Anti-obesity activity	Red yeast rice	High fat diet fed mice	Perirenal and epididymal fat pads, blood	Exhibited lower weight gain and less fat pads mass, increased the lipolysis activity of adipose tissue, and reduced food/energy consumption	4–20 g/kg	6 weeks	(Chen et al., 2008)
	Aqueous and ethanol extracts	3T3-L1 cells	3T3-L1 cells	Suppressed the proliferation and differentiation, enhanced the lipolysis activity	50–200 μg/mL	24–48 h	(Chen et al., 2008)
	Aqueous extracts	High fat diet fed mice	Mice	Prevented weight gain, reduced the mesenteric fat pad weight	1–2.5 g/kg	8–12 weeks	(Lee et al., 2015)
Anti-inflammatory activity	Red yeast rice capsule	High cholesterol diet fed mice	Kidney and serum	Reduced the expression levels of TNF−α and IL-6, and repaired kidney damage	300 mg/kg	6 weeks	(Ding et al., 2014)
	Monascusazaphilone A	RAW264.7 cells	RAW264.7 cells	Inhibited NO production	1–20 μg/mL	24 h	(Wu et al., 2013)
	Monapurfluores A and B, monascopyridines C and D, monasfluores A and B	RAW264.7 cells induced by lipopolysaccharide	RAW264.7 cells	Inhibited NO production	5–20 μg/mL	12 h	(Hsu et al., 2010)
Anti-hypertensive activity	Ethanol extracts	Spontaneously hypertensive rats	Rats and blood	Exhibited anti-hypertensive effects, decreased heart rate, cardiac contractility, and sympathetic vasomotor tone, increased plasma NO production	10–50 mg/kg	30–120 min	(Wang et al., 2010)
Immunomodulatory activity	Polysaccharides	Mice	Thymus, spleen, foot, and blood	Improve phagocytic ability of abdominal cavity macrophage, prompted the formation of periphery blood E-rose loop, increased the transformation rate of lymphocyte, and strengthened nonspecific immunity	50–300 mg/kg	7–14 days	(Zhang et al., 2008)
Improving production and quality of eggs	Red yeast rice powder	Laying hens	Blood and eggs	Increased laying rate, albumen height, haugh units, and serum calcium levels, lowered yolk cholesterol, serum cholesterol and triglyceride levels	0.5–1.5 g/kg	8 weeks	(Sun et al., 2015)
	Red yeast rice capsule	Japanese quail	Blood and eggs	Increased egg production, lowered yolk cholesterol levels	0.03–0.12 mg/g	8 weeks	(Pengnoi et al., 2018)

Dash (—) denotes no useful information found in the study. HDL-C, high density lipoprotein cholesterol; TC, total cholesterol; TG, triglyceride; LDL-C, low density lipoprotein cholesterol; SGAs, second-generation antipsychotics; XZK, Xuezhikang; LPL, lipoprotein lipase; LDLR, low density lipoprotein receptor; Hs-CRP, high sensitivity C-reaction protein; PWV, pulse wave velocity; TNF-α, tumor necrosis factor-α; MMP, metalloproteinase; NF-κB, nuclear factor-κB; HCY, homocysteine; ROS, reactive oxygen species; IL, interleukin; PSA, prostate-specific antigen; sAPPR, soluble amyloid precursor protein R-fragment; PPAR, peroxisome-proliferator-activated receptor; ALT, alanine aminotransferase; ACh, acetylcholine; 6-OHDA, 6-hydroxydopamine.

### Hypolipidemic Effect

Hyperlipidemia is the bane of current dietary patterns and rather torpid lifestyles. As a result, the search for supplements that can lower the triglyceride (TG) and cholesterol levels has gained significant momentum. Statins have been used to eliminate vascular occlusions, but with increasing reports of side effects, alternatives are being pursued (Patel, 2016). In this regard, the hypolipidemic potential of RYR has been consistently proved with reliable experimental results (Gerards et al., 2015). In dyslipidemia patients, RYR, which was administered as a dietary supplement (4–48 mg/kg), significantly decreased TG, total cholesterol (TC), and low density lipoprotein cholesterol (LDL-C) levels, and increased the high density lipoprotein cholesterol (HDL-C) level (Heber et al., 1999; Becker et al., 2009; Bruno et al., 2018). However, further studies that follow patients for longer periods of time are needed to investigate the effects RYR on the risk factors and treatment of various chronic diseases. In animals fed a high-fat diet, the administration of RYR, as a capsule, or RYR aqueous extracts significantly down-regulated TG, TC, and LDL-C levels (Wei et al., 2003). These hypolipidemic activities of RYR were mediated at least partially by enhanced acidic sterol excretion and reduced expression of inflammatory transcription factors such as tumor necrosis factor-α (TNF-α) and interleukin-6 (IL-6) (Ma et al., 2009; Ding et al., 2014). However, the mechanisms of action remain to be defined.

The putative hypolipidemic effects of RYR have been mostly ascribed to the rich contents of monacolins and pigments (Zhou et al., 2019). In a crossover, double-blind, placebo-controlled randomized clinical trial, short-term treatment (4 weeks) with monacolins (10 mg) could significantly reduce TC, LDL-C, and non-HDL levels (Cicero et al., 2013). However, these results have yet to be confirmed in studies following a larger cohort for a longer period of time. In another study that utilized a high-fat diet rat model, the administration of MK (5–30 mg/kg) decreased serum TC, TG, and LDL-C levels by up-regulating lipoprotein lipase and low density lipoprotein receptor mRNA expression in the liver (Chen, 2004). The oral administration of RYR yellow, red, and orange pigments also markedly alleviated disturbances in lipid metabolism; ameliorated serum lipid levels; suppressed hepatic lipid accumulation and steatosis; and promoted fecal cholesterol, triacylglycerol, and bile acid excretion. The mechanisms of action involve an up-regulation of the mRNA levels of farnesoid X receptor and peroxisome-proliferator-activated receptor-gamma, the main receptors for the metabolism of cholesterol and homeostasis of bile acids (Zhou et al., 2019). However, some of these results did not show a dose-effective relationship, and some of these studies lacked positive controls.

RYR is often combined with other products that can effectively reduce lipid levels, such as berberine (Millan et al., 2016), policosanols (Guardamagna et al., 2009), coenzyme Q_10_ (Cicero et al., 2016), plant stanols and sterols (Feuerstein and Bjerke, 2012), olive extracts (Verhoeven et al., 2015), and curcumin (Derosa et al., 2018). Among these, berberine and policosanols have been extensively investigated due to their lipid-lowering properties, in which an increase in LDL receptor half-life and expression and an increase in insulin sensitivity *via* the activation of AMP-activated protein kinase (AMPK) (Spigoni et al., 2017) were observed. *Bifidobacterium longum*, a probiotic strain showing potent hypolipidemic effects, has also been combined with RYR in nutraceutical formulations given that alterations in gut microbiota were found to be involved in the pathogenesis of systemic diseases related to hypercholesterolemia (Weis, 2018; Ruscica et al., 2019). Additionally, several Chinese medicines have been used with RYR to treat hyperlipidemia, such as *Tao He Cheng Qi Tang* consisting of *Prunus persica* semen, *Cinnamomum cassia* ramulus, *Rheum palmatum* rhizome, natrii sulfas, and *Glycyrrhiza uralensis* rhizome (Yang, 2017). However, the possible interactions, synergistic effects, and underlying mechanisms of RYR in combination with other ingredients from poly-herbal preparations remain unknown and should be further investigated.

### Anti-Atherosclerotic Activity

The inhibition of cholesterol synthesis is effective for the primary and secondary prevention of atherosclerotic diseases (Li et al., 2005b). Experimental studies in animal and cell models have demonstrated that RYR, as well as Xuezhikang (XZK, its pure extract), has anti-atherosclerotic activity. An eight-week administration of XZK (600–1,200 mg/kg) to C57BL/6 mice was found to significantly and dose-dependently inhibit vulnerable plaque progression, decrease the plaque area, and suppress lesional endoplasmic reticulum stress (Shen et al., 2017). In another study involving atherosclerotic rats fed a high-cholesterol diet, the oral administration of RYR at 120 mg/kg significantly reduced the plaque size, stabilized the plaque, protected the endothelium, and decreased the number of lipid droplets and cholesterol calculi, and lowered the levels of high sensitivity C-reaction protein (Hs-CRP), IL-6, and TNF-α, suggesting that the anti-atherosclerotic activity of RYR may be related to inflammatory signaling pathways (Wu et al., 2017). The administration of aqueous and ethanol extracts of RYR to homocysteine-treated human aortic smooth muscle cells (HASMCs) (1–160 µg/ml) for 24 h reduced TNF-α-induced metalloproteinase (MMP)-2 and MMP-9 expression. It also reduced nuclear factor-κB (NF-κB) activation and intracellular reactive oxygen species (ROS) formation, supporting the notion that RYR may have a potential application in the treatment of atherosclerosis (Lin et al., 2011). Unfortunately, the chemical analyses of XZK and the aqueous and ethanol extracts of RYR were not performed in these studies. Based on the significant decrease in the viability of HASMCs treated with relatively high concentrations of RYR (160 µg/ml), attention should be paid to the possible side-effects in clinical applications.

### Anti-Cancer Activity

Cancer, a major disease, is the leading cause of death throughout the world, and developing effective anti-cancer therapies remains a challenge for those in medical research (Zhu et al., 2018). Previous studies have reported that RYR supplements have anti-cancer activity. The oral administration of RYR (120 mg/kg, qd) to breast cancer patients for 12 weeks significantly decreased serum tumor biomarker (CA125, CA153) levels and improved their life quality and immune function after chemotherapy and resection surgery (Zhong, 2017). Although this study showed that RYR possesses anti-cancer activity, only a single dose was used, thereby limiting information on the dose-dependent effect. In SCID tumor mice induced by human prostate cancer cells, the administration of powdered RYR significantly reduced the oral tumor volume and decreased the serum prostate-specific antigen level, as well as the gene expression of enzymes involved in androgen synthesis (HSD3B2, AKR1C3, and SRD5A1) (Hong et al., 2011). Thus, clinical studies of RYR use for the prevention of prostate cancer in men undergoing active surveillance of the disease should be considered.

Studies have reported that polysaccharides, monacolins, and pigments from RYR are responsible for the anti-cancer activity of RYR. The administration of RYR polysaccharides (at a very high dose of 800 mg/kg) to replanted S_180_ tumor mice for 2 weeks remarkably inhibited tumor growth and improved body weight (Ding, 2007). However, this trial lacked a positive control as well as an evaluation of serum biochemical indicators of the tumor. In K-562, SK-OV-3, and SNU-1 cells, the administration of rubropunctatin, a red pigment, and RYR-derived monacolin L (1.25–40 µg/ml) for 48 h exhibited very strong inhibitory effects against cancer cell proliferation, and the effect of rubropunctatin was comparable with that of anti-cancer drugs *cis*-platinum, taxol, and 10-hydroxy-camptothecin in their IC_50_ values. In this study, the authors demonstrated that RYR at least partially exerted its anti-cancer effects through telomerase-mediated inhibition of rubropunctatin and monacolin L-induced apoptosis (Xu et al., 2017). However, both studies failed to discuss the effectiveness of RYR-derived monacolin and pigments using animal models, and more in-depth studies should be carried out to further develop these compounds into anti-cancer drugs.

### Neurocytoprotective Activity

RYR extracts have also been shown to possess neurocytoprotective activity. In rats treated with amyloid β (Aβ), an amino acid associated with Alzheimer’s disease, the oral administration of RYR (151–755 mg/kg) for 4 weeks could markedly reverse the memory deficits in the water maze and passive avoidance tasks, as well as prevent Aβ40 infusion and damage in the hippocampus and cortex, which are known involve in the increase of thiobarbituric acid reactive substances and ROS. Using human neuroblastoma IMR32 cells exposed to a high concentration of cholesterol, the authors also reported that RYR down-regulated Aβ40 formation and deposition by suppressing cholesterol-induced β-secretase activity and apolipoprotein E expression. RYR also mediated the proteolysis of amyloid precursor protein (APP) into a soluble APP α-fragment with neuroprotective activity in the hippocampus (Lee et al., 2010). However, further studies are needed to confirm the neurocytoprotective effect using low doses of RYR suitable for human administration. Lovastatin derivatives are also responsible for the neurocytoprotective effect of RYR. The administration of one lovastatin-derived compound, designated **3f** (12.5–100 µmol/L), for 24 h significantly reduced 6-hydroxydopamine (6-OHDA)-induced apoptosis in PC12 cells, reduced caspase-3, -8, and -9 activities, and lowered intracellular calcium concentrations elevated by 6-OHDA in a concentration-dependent manner, without inhibiting the production of ROS (Lin et al., 2015). Studies on the mechanism of action of compound **3f** are being conducted.

### Hepatoprotective Effect

RYR extracts exhibit a strong hepatoprotective effect both in alcoholic fatty liver and non-alcoholic fatty liver disease (NAFLD) mice models. In chronic alcohol-induced mice, the oral administration of powdered RYR (307.5–1,537.5 mg/kg) for 5 weeks significantly attenuated the increased levels of serum transaminases (aspartate aminotransferase and alanine aminotransferase), hepatic TGs, and TC. Furthermore, RYR elevated the hepatic antioxidant activity that reduced hepatic cell damage (steatosis) and decreased tissue inflammatory cytokine levels (Cheng and Pan, 2011). These findings suggested that RYR may represent a novel, protective strategy against alcoholic liver disease by attenuating oxidative stress, inflammatory responses, and steatosis. However, the authors of this study did not perform comparisons using a positive control. The administration of RYR extracts (XZK at 300 mg/kg) to NAFLD mice for 6 weeks significantly ameliorated dyslipidemia and fat accumulation in the liver; improved insulin resistance; ameliorated oxidative stress; lessened hepatic steatosis, necro-inflammation, and collagen deposition; and reversed abnormalities in the aminotransferase level. The mechanism of action of RYR likely involves inhibition of the hepatic expression of TNF-α (Hong et al., 2007). These results suggested that RYR may have a potential clinical application in the treatment of NAFLD. In this study, however, the optimal dose, constituents, and side effects of RYR were not assessed.

### Anti-Osteoporotic Activity

Osteoporosis is a common health problem characterized by low bone mass and structural deterioration of the bone, resulting in an increased susceptibility to fractures (Zhang et al., 2018b). Several studies have reported the protective role of RYR in osteoporosis. In rats with ovariectomy-induced bone loss and osteoblast cells, the administration of ethanol extracts from RYR (at a very high dose of 1.56–6.24 g/kg) for 20 weeks significantly increased the bone mineral density and decreased the levels of bone turnover markers *in vivo*, including osteocalcin and tartrate-resistant acid phosphatase. Moreover, its administration improved the viability of osteoblasts and enhanced the mRNA and protein expression of bone morphogenetic protein (Bmp) 2 and Bmp4, suggesting that RYR may be useful in preventing and treating osteoporosis (Wang et al., 2015). However, future studies should confirm this anti-osteoporotic effect under clinical conditions with low doses of RYR that are suitable for human administration. In rabbits with bone defects and UMR 106 cells, the administration of RYR extracts significantly promoted new bone formation *in vivo*, enhanced bone optical density, and increased alkaline phosphatase activity *in vitro*, suggesting that RYR is a natural product that can potentially treat bone defects and osteoporosis (Wong and Rabie, 2008). However, additional evidence from randomized controlled trials is required to identify other regulatory mechanisms that may be responsible for the anti-osteoporotic effects. The bioactive constituents of these extracts also remain unknown.

### Anti-Fatigue Activity

RYR extracts exhibit strong anti-fatigue capability. The oral administration of RYR (24 mg/kg) to dyslipidemia patients for 4 weeks was found to significantly reduce muscle fatigue and preserve physical activity (Xue et al., 2017). Nevertheless, only a single dose of RYR was used in this study. In a Wistar rat model, the administration of powdered RYR (at a very high dose of 1–5 g/kg) for 4 weeks significantly extended the swimming time for the rats, effectively delayed the lowering of the glucose level in the blood, prevented the increase in lactate and blood urea nitrogen concentrations, and decreased the contribution of exercise-induced oxidative stress, suggesting that RYR has anti-fatigue activity and may potentially be a useful pharmacological agent (Wang et al., 2006). However, the main shortcomings of this study were that the dose of RYR was too high and no mechanism of action was provided. RYR extracts are abundant monacolins and pigments. The administration of MK (10–90 mg/kg) or ethanol-soluble red pigments (50–200 mg/kg) to mice for 30 days significantly improved the hepatic glycogen level, decreased the serum urea nitrogen level, and increased the swimming time in an endurance test, indicating that MK and red pigments are responsible for the anti-fatigue activity of RYR (Chen, 2004). However, future studies are needed to identify the mechanism of action of these constituents.

### Anti-Diabetic Activity

Diabetes is assuming epidemic proportions across the world (Lam and LeRoith, 2012). The best approach to control hyperglycemia and mitigate diabetes is dietary intervention, rather than a reliance on drugs (Patel, 2016). In this regard, the possible role of RYR was investigated. The administration of RYR (50–350 mg/kg) to streptozotocin-induced diabetic rats for 2 weeks markedly decreased the plasma glucose level, reversed hyperphagia, and decreased the mRNA level of phosphoenolpyruvate carboxykinase in the liver in a dose-dependent manner, indicating that RYR decreased hepatic gluconeogenesis and lowered the plasma glucose level in diabetic rats (Chang et al., 2006). A similar study has reported that RYR could promote the release of acetylcholine from nerve terminals, which in turn stimulated muscarinic M3 receptors in pancreatic cells and augmented insulin release in order to lower the plasma glucose level (Chen and Liu, 2006). In another study, the administration of RYR (300 mg/kg) to diabetic mice for 8 weeks significantly decreased the blood glucose level by improving glucose tolerance and insulin secretion, protected islets from hyperglycemic injury, and inhibited the expression of key factors in oxidative stress, including 8-OHdG, 4-HNE, and gp91phox, indicating that the effects of RYR on oxidative stress may at least partly account for the improved insulin secretion of pancreatic islets in diabetes (Wang et al., 2014). However, this evidence is still tenuous; no detailed clinical trials involving RYR supplementation have been performed, and no chemical constituents of RYR responsible for the anti-diabetic activity have been presented. Furthermore, several studies also failed to include positive controls and dose-dependent effect analyses.

### Anti-Obesity Activity

Obesity is defined as an excess of white adipose tissue, which is associated with a higher risk of developing diabetes and cardiovascular disease (Marques et al., 1998). RYR has been shown to possess anti-obesity activity. In mice fed a high-fat diet, the administration of RYR (at a very high dose of 4–20 g/kg) significantly lowered weight gain and fat pad mass, which was accompanied by smaller fat cells. The anti-obesity effects of RYR were mainly derived from the lipolytic activity and mild anti-appetite potency of RYR. In 3T3-L1 cells, the investigators demonstrated that RYR extracts suppressed the proliferation and differentiation of preadipocytes, which may have inhibited new adipocyte formation or hyperplasia in adipose tissue (Chen et al., 2008). The main shortcoming in this study was the very high dose of RYR, which will restrict the clinical application of RYR in the treatment of obesity.

### Anti-Inflammatory Activity

Inflammation signifies an agitated metabolic and immunological system (Odegaard and Chawla, 2013), and anti-inflammatory drugs that can restore the homeostasis of a perturbed system are in great demand. Various anti-inflammatory drugs exist, although many associate with adverse effects (Patel, 2016). RYR has been reported to possess an inflammation-quenching effect. In RAW264.7 cells induced by lipopolysaccharide, the administration of azaphilonoid derivatives isolated from RYR (5–20 µg/ml), including monapurfluores A and B, monascopyridines C and D, and monasfluores A and B, significantly inhibited the release of the inflammatory mediator nitric oxide (NO) from macrophages (Hsu et al., 2010). NO has been reported to be a signaling agent for various inflammatory diseases; therefore, its suppression is likely to ameliorate irritation (Patel, 2016). However, this study failed to include a positive control, and other inflammatory indicators, such as IL-1β, TNF-α, and IL-6, should be studied in the future. In mice fed a high-cholesterol diet, the administration of XZK (300 mg/kg) for 6 weeks significantly reduced the levels of the inflammatory transcription factors TNF-α and IL-6, and attenuated renal injury by managing the abnormal lipid levels and the consequent stress (Ding et al., 2014). This study also lacked a positive control, and the effects of the various doses were not assessed.

### Anti-Hypertensive Activity

Hypertension, commonly recognized as a silent killer, is the most common cardiovascular disease and a major risk factor for atherosclerosis, metabolic syndrome, renal dysfunction, myocardial infarction, heart attack, and stroke, which are the most prevalent causes of death in industrialized countries (Williams, 2009; Wang et al., 2010). As many drugs have side effects, healthy diets are expected to prevent or alleviate the complications. The potential of RYR, in combination with other bioactive components, to manage the problem is discussed below. In grade 1 essential hypertension patients, the administration of a nutraceutical formulation containing RYR, folic acid, coenzyme Q_10_, *Orthosiphon stamineus* (a tropical herb from the Lamiaceae family), policosanol, and berberine significantly reduced the mean 24-h systolic and diastolic blood pressure levels (Trimarco et al., 2012). However, the ratios between RYR and the other constituents are not optimized based on the anti-hypertensive activity. In spontaneously hypertensive rats, the intravenous bolus administration of RYR ethanol extracts (10–50 mg/kg) elicited biphasic and potent hypotensive and cardiac inhibitory effects, as well as a significant decrease in sympathetic vasomotor activity (Wang et al., 2010). Further studies are needed to identify the chemical constituents in RYR ethanol extracts responsible for the anti-hypertensive effects. Meta-analysis of the existing literature showed that RYR, together with conventional therapy and statins, can lower the systolic blood pressure, without any significant adverse side effects (Patel, 2016; Xiong et al., 2017). Taken collectively, the results are encouraging but limited. Although meaningful reductions in blood pressure have been observed, evidence for the use RYR in the treatment of hypertension is weak based on the available data. Thus, more rigorous, high-quality trials are required to provide stronger evidence.

### Other Activities

Polysaccharides from RYR have been shown to possess immunomodulatory activity. In a mice model, the administration of RYR-derived polysaccharides (50–300 mg/kg) significantly improved the phagocytic activity of abdominal cavity macrophages, prompted the formation of the peripheral blood E-rose loop, increased the transformation rate of lymphocytes, and enhanced nonspecific immunity (Zhang et al., 2008). However, this study failed to include a positive control, and the relationship between polysaccharide structure and immunomodulatory activity should be further investigated. In laying hens, the administration of RYR significantly increased the laying rate, Haugh units, and albumen height, as well as decreased the yolk cholesterol content, suggesting that RYR plays important roles in improving the production and quality of eggs (Sun et al., 2015). Further studies are required to determine the level of citrinin, a nephrotoxin in RYR, in the serum and eggs of hens in order to meet a lower citrinin level with regard to RYR production.

Methane (CH_4_), a greenhouse gas that is 25 times more effective than carbon dioxide in global warming, is emitted during enteric fermentation in ruminants, accounting for approximately 10% of the total anthropogenic CH_4_ (Holter and Young, 1992; Hristov et al., 2013). In a castrated Boer crossbred goat model, the administration of RYR significantly reduced enteric CH_4_ emissions, serum lipid levels, and the growth of several archaeal species (e.g. *Methanobrevibacter*) in the rumen. Therefore, as a natural fermentation product, RYR can reduce enteric methane emissions in goats (Wang et al., 2016). However, caution should be taken when considering the digestibility of protein and organic matter, and further studies are needed to evaluate its effects in different animals with various diets as well as its effects on animal health and food safety.

## Pharmacokinetic Studies

MK (also known as lovastatin), categorized as a class II compound according to the Biopharmaceutics Classification System (BCS), is mainly responsible for the blood cholesterol lowering effect of RYR (Wu and Benet, 2005). MK exhibits poor oral bioavailability (<5%) because of its low solubility (1.3 µg/mL in water), extensive metabolism in the gut and liver, and transmembrane efflux *via* the drug transporter P-glycoprotein (Serajuddin et al., 1991; Chen et al., 2005). Its bioavailability can be improved by increasing the dissolution rate and/or decreasing pre-systemic clearance (Neuvonen et al., 2008; Wu et al., 2011). In Caco-2 cells, extracts of RYR (LipoCol Forte, XZK, and Cholestin) were more effective in inhibiting the activities of CYP450 enzymes and P-glycoprotein, and showed higher lovastatin absorption and dissolution rates than that of lovastatin alone (Chen et al., 2012a; Chen et al., 2013). Moreover, in human studies, the authors demonstrated that volunteers receiving LipoCol Forte capsules or powder exhibited higher area under the plasma concentration-time curve and C_max_ (maximum plasma concentration) values for both lovastatin and its active metabolite, lovastatin acid, and lower T_max_ (time to reach the peak concentration) values than those receiving lovastatin tablets, suggesting that the oral bioavailability of lovastatin is significantly improved when combined with RYR as a result of the higher dissolution rate (Chen et al., 2013). In this regard, the increased dissolution rate seen with RYR products might enable lovastatin (a BCS class II drug) to function as a BCS class I drug. Additionally, Leone and colleagues (Leone et al., 2016) prepared a new formulation that combined 60% gelatin with 40% alginate. They observed a delayed release of lovastatin from RYR, a prolonged inhibition of 3-hydroxy-3-methylglutaryl-coenzyme A reductase, and decreased cholesterol synthesis. However, further studies are needed to explore the underlying mechanism of actions, as well as the pharmacokinetics of the other bioactive compounds present in RYR, including monacolins and pigments such as rubropunctamine, monascorubramine, and ankaflavin.

## Quality Control

To assess and control the quality of RYR, the Chinese Pharmacopoeia suggests using morphology, ultraviolet-visible spectrophotometry, thin-layer chromatography, and high-performance liquid chromatography (HPLC). MK can be used as a marker of quality in the official monograph of the RYR as well as in the label claim of several commercial RYR-related products. The MK content of RYR should be more than 0.22% by HPLC (Chinese Pharmacopoeia, 2015). However, the European Commission has not legislated on the limits for RYR supplements and there is no standardization in European countries, while the Food and Drug Administration (FDA) has stated in 2007 that RYR products containing MK are identical to a drug, and therefore, subject to regulation (FDA, U.S, 2007). With regard to therapeutic efficacy, a 2011 European Food Safety Authority (EFSA) report confirmed that the daily intake of 10 mg of MK was beneficial in maintaining normal cholesterol levels (EFSA, 2011). However, manufactures of RYR products rarely disclose the levels of MK and the active ingredients are not standardized.

Various methods are available for the determination of the MK level in RYR products, including HPLC (Theunis et al., 2017), ultra-high-performance liquid chromatographic with diode array detection and/or with mass spectrometry (UHPLC–DAD–QToF-MS) (Avula et al., 2014), matrix effect-free MISPE-UHPLC-MS/MS (Svoboda et al., 2017), LC-DAD/FLD (fluorescence detection)-MS^n^ (Mornar et al., 2013), UHPLC hyphenated with triple quadrupole tandem MS (UHPLC–QQQ-MS) (Zhu et al., 2013), flow injection FI-MS/MS (Song et al., 2012), square-wave voltammetry (Nigovic et al., 2015), and contact angle, atomic force microscopy with Fourier transform infrared spectroscopy (Eren et al., 2015). However, the use of only one quantitative marker may be insufficient to properly assess the quality of RYR extracts. There is evidence to indicate that the pigments found in RYR have hypolipidemic (Zhou et al., 2019), anti-cancer (Xu et al., 2017), anti-fatigue (Chen, 2004), and anti-inflammatory (Hsu et al., 2010) activities, and therefore, the presence of pigments may be a critical indicator of extract quality. Although several pigments, such as rubropunctatin, monascorubrin, monascin, monasfluore A, and monasfluore B, have been identified in RYR by liquid chromatography–mass spectrometry (LC-MS) (Jin et al., 2016), HPLC-FLD (Huang et al., 2011), and HPLC-UV (Huang et al., 2016), more rapid, sensitive, and selective technologies should be developed for the detection of pigments.

In RYR extracts, the content of active ingredients, which includes MK, pigments, phenolic compounds, and flavonoids, varies in quality with the fermentation process, extraction procedures used, strain of *M. purpureus*, and storage conditions. The MK concentration in RYR can be increased by the mixed-culture fermentation of rice with two different Monascus species (*M. purpureus* and *M. ruber*), with the optimized fermentation process parameters including a pH of 6.03 at a temperature of 29.46°C for 13.89 days. This process predicts 2.83 mg/g and yields 2.80 mg/g of MK/gram of fermented rice with 98.93% accuracy (Panda et al., 2010). Moreover, the supplementation of the fermentation substrate with nitrogen sources influences the pigment production efficiency of RYR. For instance, the addition of orange peels and sunflower meal to solid-state fermentation (SSF) cultures of *M. purpureus* can enhance pigment production in RYR, yielding 9 absorbance units (AU)/per g of dry fermented substrate (gdfs) (Kantifedaki et al., 2018). Babitha and colleagues (Babitha et al., 2007) reported that the addition of monosodium glutamate to jackfruit seed SSF of *M. purpureus* improved the production of red (30.8 AU/gdfs) and yellow (25.5 AU/gdfs) pigments, followed by the addition of soybean meal, peptone, and chitin powder. In addition, a combination of light and bacteria can be used to enhance secondary metabolite production during RYR fermentation. The fermentation of RYR with *Bacillus subtilis*, a rod-shaped, Gram-positive endospore-forming bacterium, under blue light-emitting diodes enhanced the production of phenolic compounds (68.4 ± 1 mg GAE/g DW) and flavonoids (51.7 ± 1 mg QE/g DW) compared to white light and darkness (Elumalai et al., 2019). Further studies should focus on the molecular mechanisms and optimization of conditions for the commercial-scale production of these secondary metabolites. Under optimal extraction conditions with 45% ethanol, 1.5% phosphate, and an extraction time of 70 min, 91.6% of citrinin was removed and 79.5% of MK was retained in the final RYR extract (Lee et al., 2007a). *M. purpureus* strains are commonly used to enhance the production of MK and pigments in RYR. Using rigorous physicochemical mutagenesis technologies involving ultraviolet-lithium chloride, microwave heating, and genome shuffling mutations, two mutant *M. purpureus* strains, strain 183-3 with a highly stable capacity to produce pigment and strain R”-30 with a highly stable capacity to produce MK, were obtained (Xu, 2014; Li, 2016). In addition, Li et al. reported that the MK content in RYR significantly decreased under conditions of high humidity, high temperature (75% RH, 60°C), and sunlight, indicating that MK in RYR is light- and heat-sensitive, and RYR should be stored in the cool and dark place (Li et al., 2005a).

The area in which RYR is produced also influences its quality. RYR is now produced in more than 120 cities in 15 countries in Asia, Europe, and North America, such as China, Japan, the United States, Germany, Italy, Thailand, India, Korea, and Belgium. The general geographical distribution of RYR is shown in **Figure S2**. In studies that used UHPLC–DAD–QToF-MS to determine the MK content in 26 brands of RYR from four large retail chains in the United States (i.e. GNC, Walgreens, Walmart, and Whole Foods), it was reported that the MK content was highly variable, ranging more than 60-fold from 0.09 to 5.48 mg per 1,200 mg (Cohen et al., 2017). In another study that involved LC-PDA-MS, Huang and colleagues found that RYR from cities in Fujian Province, especially *Gutian*, *Nanping*, and *Pinnan*, possessed a higher MK content compared to six other habitats in China (Huang et al., 2006). These results indicate that the MK content in RYR extracts significantly differs between samples from different habitats. Thus, the MK content should be compared across RYR products, uniform legislation should be proposed, and strict quality control of RYR extracts should be implemented.

Citrinin is a polyketide secondary metabolite found in food and feed that is produced by several fungi, including *M. purpureus* (Bezeria da Rocha et al., 2014; Kiebooms et al., 2016). It is a mycotoxin known to cause kidney damage as well as disrupt metabolic processes in the liver (Mornar et al., 2013). Citrinin, as a component, has been involved in several controversies related to the quality and safety of RYR products, because up to 80% of RYR products may contain this mycotoxin (Heber et al., 2001). The EFSA has set a level of no concern for citrinin-caused nephrotoxicity at 0.2 µg/kg body weight per day, but it has acknowledged the need for more reliable data on the citrinin level in food and feed before it can undertake exposure studies and risk assessment (Marley et al., 2016). Different analytical methods have been developed for the quantification of citrinin in a variety of samples in the last few years, including LC-FDA (Marley et al., 2016), UHPLC-MS/MS (Kiebooms et al., 2016), UHPLC–DAD–QToF-MS (Avula et al., 2014), LC-DAD/FLD-MS^n^ (Mornar et al., 2013), microsphere-based flow cytometric immunoassays (MFCI) (Li et al., 2012), and a multi-commutated fluorometric optosensor technology (Jimenez Lopez et al., 2014). The maximum allowed level of citrinin as a contaminant in RYR is 200 ppb in Japan, but the European Union has recommended a limit of 100 ppb (Mornar et al., 2013; Nigovic et al., 2013). However, many commercial RYR products do not meet the criteria, and the highest citrinin concentration detected by HPLC was 140 mg/kg (Ji et al., 2015). More in-depth studies are necessary to arrive at more accurate conclusions and to extrapolate data for risk assessment with respect to the prevalence of citrinin in our food chain and its potential impact on human health.

## Safety and Adverse Effects

According to the currently available results of *in vitro* and *in vivo* studies, RYR does not seem to be no mutagenic or toxic. In an albino rat model, the administration of acute doses of RYR at 0.5–5.0 g/kg body weight did not cause toxicity or mortality. Similarly, the administration of RYR at a level of 2.0–12.0% (w/w) for 14 weeks did not produce any significant changes in the food intake or body weight of the treated rats compared to control rats (Kumari et al., 2009). In an Ames test, the equivalent of up to 1 mg of ethanol extract from RYR per plate exhibited no genotoxicity toward *Salmonella typhimurium* strains TA 98, TA 100, and TA 102. Moreover, it has been reported that high levels of RYR exhibited no toxicity, following subchronic administration at levels up to 1,000 mg/kg of body weight for 28 and 90 consecutive days in the rats (Yu et al., 2008).

However, RYR does have adverse effects because MK and citrinin associate with an increased risk of myopathy (Lapi et al., 2008; Polsani et al., 2008; Dobremez et al., 2018), symptomatic hepatitis (Roselle et al., 2008), peripheral neuropathy (Kumari et al., 2013), erectile dysfunction (Liu and Chen, 2018), and anaphylactic reactions (Hipler et al., 2002). Among these, the prevalence rates of myopathy and hepatitis are the highest. Using Italy’s WHO-UMC system, CIOMS/RUCAM score, and WHO-Vigibase, 52 out of 1,261 studies reported 55 adverse reactions to RYR dietary supplements from April 2002 to September 2015, and myopathy and hepatitis accounted for 52.73% of the total adverse reactions (Mazzanti et al., 2017). Although RYR supplementation appears promising for patients with hypercholesterolemia or hyperlipidemia or those at high risk for cardiovascular events, there is insufficient regulation of RYR-containing supplements in China, the United States, and European countries. Until there is a standardized system in place for product formulation and manufacturing, and the amount of MK in a given RYR supplement is clearly stated, the effectiveness and safety of these products will remain in question (Dujovne, 2017; Peng et al., 2017). Thus, real-world vigilance should be used, and it should include consumers, clinicians, and policymakers to promote the proper use of RYR. Furthermore, randomized controlled trials should be carried out to test the safety and efficacy of each RYR preparation.

The safety profile of RYR supplements is highly similar to that of statins (Mazzanti et al., 2017). Some statins, including lovastatin (MK in RYR), are metabolized by the cytochrome P450 enzyme CYP3A4 and it is known that coadministration of drugs that inhibit CYP3A4 can increase the plasma half-lives of these statins and the risk of myotoxicity (Mastaglia and Needham, 2012). CYP3A4 inhibitors reported to interact with statins and to cause rhabdomyolysis include clarithromycin (Grunden and Fisher, 1997), verapamil (Choi et al., 2010), cyclosporine (Olbricht et al., 1997), diltiazem (Azie et al., 1998), itraconazole (Neuvonen and Jalava, 1996), and azithromycin (Grunden and Fisher, 1997). Statin-induced rhabdomyolysis may result from increased exposure to the drug, plausible mechanisms for the interaction include increased absorption of lovastatin by competition at gut P-glycoprotein or decreased excretion of statin due to competition for renal tubular secretion or interaction at hepatic CYP3A4 sites (DiGregorio and Pasikhova, 2009). Co-administration of a statin and a fibrate drug also increases the rhabdomyolysis risk, and gemfibrozil in particular should be avoided in patients receiving lovastatin (Franssen et al., 2009). Therefore, the healthcare providers need to heighten their awareness and screening for potential drug-drug interaction in patients consuming RYR products, and consider early monitoring of liver function and signs of muscle injury. In parallel, consumers should be informed that MK contained in RYR is identical to lovastatin, and need to be discouraged from using RYR preparations as self-medication, particularly if they have experienced previous adverse reactions to statins.

## Conclusions and Future Perspectives

RYR is widely used in prescriptions, as well as an alternative medicine and a food supplement, in Asia, the United States, and European countries. This review summarized the current information on the traditional uses, chemistry, pharmacology, pharmacokinetics, quality control, and toxicity of RYR. In classical Chinese herbal textbooks and the Chinese Pharmacopoeia, RYR is commonly used for resolving turbidity, invigorating blood circulation, and resolving blood stasis. Pharmacological studies have revealed that RYR possesses many biological properties with hypolipidemic, anti-atherosclerotic, anti-cancer, neurocytoprotective, hepatoprotective, anti-osteoporotic, anti-fatigue, anti-diabetic, anti-obesity, immunomodulatory, anti-inflammatory, anti-hypertensive, and anti-microbial activities. The results of these pharmacological investigations support the traditional use of RYR. Furthermore, more than 101 compounds have been isolated from RYR. Among these, monacolins and pigments are the most abundant and the major bioactive constituents in RYR.

Outstanding progress has been made in the chemistry and pharmacology of RYR. However, several gaps in knowledge will require additional studies. Firstly, the systematic data on the toxicology and drug interactions of RYR extracts are limited, and there are only a few studies documenting the toxic effects of RYR. Presently, the evidence is insufficient for RYR to be interpreted as toxic. Thus, a comprehensive toxicological study of both the bioactive extracts and isolated constituents from RYR is urgently needed. Furthermore, due to its potential *in vitro* and *in vivo* toxic effects, the clinical application of RYR should be restricted until more definitive studies demonstrate its safety, quality, and efficacy. Secondly, although monacolins in RYR have been found to possess pharmacological activities that are similar to those in pigments, such as hypolipidemic activity, the mechanisms underlying the absorption, distribution, metabolism, and excretion, as well as the synergistic or antagonistic effects between the two constituents, are unknown; thus, they should be further studied. Moreover, other bioactive constituents of RYR, such as organic acids, sterols, decalin derivatives, and flavonoids, should also be examined for their potential use in the development of drugs and as a food supplement. Thirdly, well-designed pharmacodynamic and pharmacological studies should be designed, and comprehensive investigations should be conducted to identify the relevant compounds responsible for the pharmacological effects and the potential mechanisms. Fourthly, although the pharmacological properties support the traditional uses of RYR in the treatment of blood circulation stasis, bone defects, and limb weakness, additional pharmacological studies are needed to address its use in the remediation of indigestion and diarrhea. Finally, the possible interactions, safety profile, synergistic or antagonistic effects, and underlying mechanisms between the bioactive compounds present in RYR and other combined used products remain unknown and should be further studied.

In conclusion, future studies should focus on the absorption, distribution, metabolism, and excretion of monacolins and their interactions with pigments, which would promote our understanding of the underlying mechanisms of the various biological activities of RYR. Further research should also consider the pharmacological activities that were overlooked with regard to the traditional uses of RYR, especially in the treatment of gastrointestinal diseases, such as indigestion and diarrhea. Finally, more efforts are needed in order to gain new insights into the toxicological effects of the main active compounds and the quality control of RYR based on its diverse chemical constituents.

## Data Availability Statement

The raw data supporting the conclusions of this manuscript will be made available by the authors, without undue reservation, to any qualified researcher. 

## Author Contributions

BZ, JH, and LQ conceived the review. BZ, GY, and FQ wrote the manuscript. JW collected the literatures. QZ edited the manuscript. All the authors have seen and agreed on the finally submitted version of the manuscript.

## Funding

This work was financed by the National Natural Science Foundation of China (81673528 and 81872953).

## Conflict of Interest

Author GY was employed by company Hangzhou Twin-horse Biotechnology Co., Ltd.

The remaining authors declare that the research was conducted in the absence of any commercial or financial relationships that could be construed as a potential conflict of interest.

## Abbreviations

RYR, red yeast rice; TCM, Traditional Chinese Medicine; MK, monacolin K; EBV-EA, Epstein-Barr virus early antigen; TG, triglyceride; TC, total cholesterol; LDL-C, low density lipoprotein cholesterol; HDL-C, high density lipoprotein cholesterol; TNF-α, tumor necrosis factor-α; IL, interleukin; LPL, lipoprotein lipase; LDLR, low density lipoprotein receptor; PPARγ, peroxisome-proliferator-activated receptor-gamma; AMPK, AMP-activated protein kinase; XZK, Xuezhikang; Hs-CRP, high sensitivity C-reaction protein; HASMCs, human aortic smooth muscle cells; MMP, metalloproteinase; NF-κB, nuclear factor-κB; ROS, reactive oxygen species; PSA, prostate-specific antigen; Aβ, amyloid; APP, amyloid precursor protein; 6-OHDA, 6-hydroxydopamine; NAFLD, non-alcoholic fatty liver disease; Bmp, bone morphogenetic protein; ALP, alkaline phosphatase; Ach, acetylcholine; NO, nitric oxide; CH4, methane; BCS, Biopharmaceutics Classification System; Cmax, maximum plasma concentration; Tmax, time to reach the peak concentration; HPLC, high-performance liquid chromatography; FDA, Food and Drug Administration; EFSA, European Food Safety Authority; UHPLC, ultra-high-performance liquid chromatographic; DAD, diode array detection; MS, mass spectrometry; FLD, fluorescence detection; SSF, solid-state fermentation; AU, absorbance units; gdfs, g of dry fermented substrate; MFCI, microsphere-based flow cytometric immunoassays; ACC, acetyl-CoA carboxylase.

## Supplementary Material

Figure S1 The fermentation **(A)** and commercial product **(B)** of RYR; Morphological characteristics of M. purpureus on Potato Dextrose Agar medium, front **(C)** and back **(D)**; Microstructure of M. purpureus under the optical **(E)** and scanning electron microscopes **(F)**, the arrows denote stromata of M. purpureus.

Figure S2 The general geographical distribution of RYR.

Tablel S1 Examples of traditional Chinese medicine prescriptions containing red yeast rice.
