# Supplementary figures and images for: Red Yeast Rice: A Systematic Review of the Traditional Uses, Chemistry, Pharmacology, and Quality Control of an Important Chinese Folk Medicine
Source: Front Pharmacol. 2019 Dec 2;10:1449. doi: 10.3389/fphar.2019.01449 (PMC6901015; doi:10.3389/fphar.2019.01449)

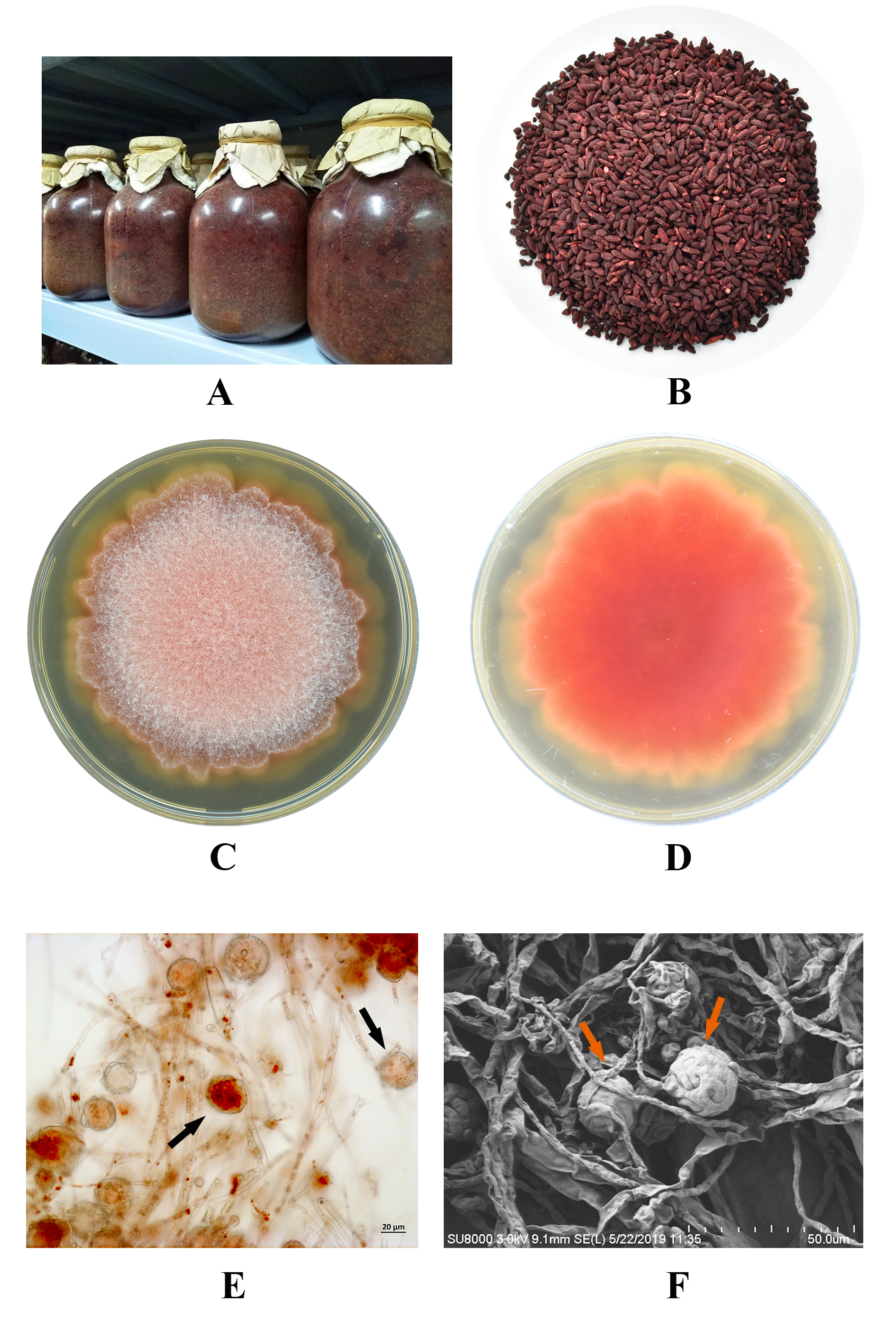

Supplement: Figure S1 — The fermentation (A) and commercial product (B) of RYR; Morphological characteristics of M. purpureus on Potato Dextrose Agar medium, front (C) and back (D); Microstructure of M. purpureus under the optical (E) and scanning electron microscopes (F), the arrows denote stromata of M. purpureus. [file Image_1.jpeg]

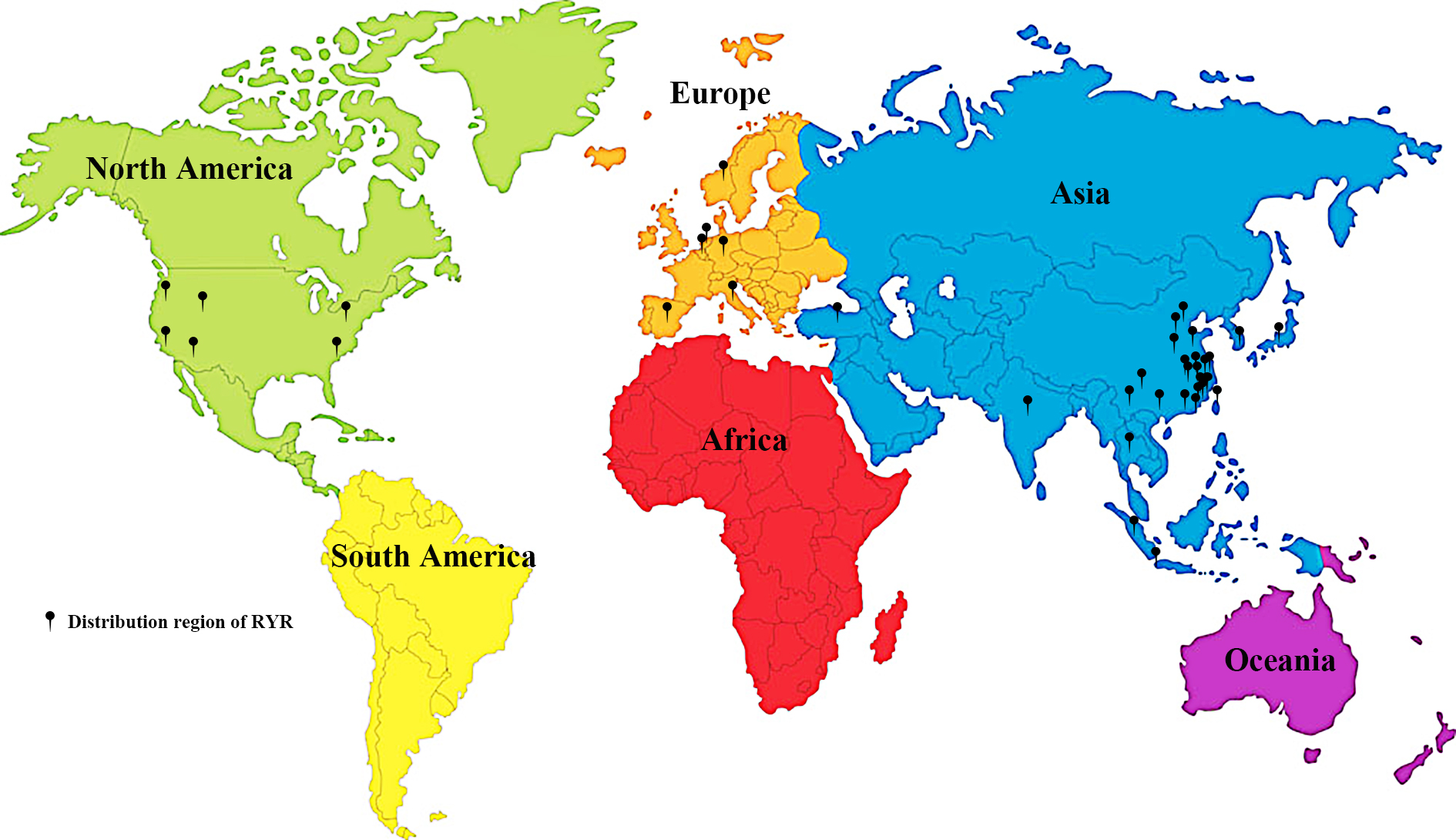

Supplement: Figure S2 — The general geographical distribution of RYR. [file Image_2.jpeg]
