# Supplementary material for: Red Yeast Rice: A Systematic Review of the Traditional Uses, Chemistry, Pharmacology, and Quality Control of an Important Chinese Folk Medicine
Source: Front Pharmacol. 2019 Dec 2;10:1449. doi: 10.3389/fphar.2019.01449 (PMC6901015; doi:10.3389/fphar.2019.01449)
Supplement: Tablel S1 — Examples of traditional Chinese medicine prescriptions containing red yeast rice. [file Table_1.docx]

Table S1 Examples of traditional Chinese medicine prescriptions containing red yeast rice

| Preparation name | Composition | Role of RYR in prescription | Traditional and clinical uses | Reference(s) |
| --- | --- | --- | --- | --- |
| Hong Qu Jiu | Red yeast rice | Leading role | Treat postpartum blood stasis | Compendium of Herbology (《本草纲目》), 1552–1578 |
| Xue Zhi Kang Pian | Red yeast rice | Leading role | Treat hyperlipemia, fatigue, vertigo, cephalalgia, diarrhea, gastrectasia | Chinese Pharmacopoeia, 2015 |
| Xue Zhi Kang Jiao Nang | Red yeast rice | Leading role | Treat hyperlipemia, fatigue, vertigo, cephalalgia, diarrhea, gastrectasia | Chinese Pharmacopoeia, 2015 |
| Bi Ma Wan | Red yeast rice, *Ricinus communis* seed | Leading role | Treat stuck throat | Imperial Medical Encyclopedia (《圣济总录》), 1111–1117 |
| Huo Tui Hong Qu San | Red yeast rice, aged ham bone power, pine pollen | Leading role | Treat deficiency of spleen, diarrhea | Following Traditional Customs Medical (《医学从众录》), 1845 |
| Liu Xing Chuan | Red yeast rice, *Litsea cubeba*, *Schizaea digitata* | Leading role | Treat smolder, hemafecia | The Continuance of Treatises Continuation on Internal and External Folk Medicine (《串雅补》), 1759 |
| Jing Tian Hua San | Red yeast rice, *Hylotelephium erythrostictum*, mirabilite | Leading role | Treat wind-toxin of spleen and lung, pruritus, and restlessness | Imperial Medical Encyclopedia (《圣济总录》), 1111–1117 |
| Huang Lian Hong Qu Tang | Red yeast rice, *Coptis chinensis*, *Scutellaria baicalensis*, *Paeonia lactiflora*, *Glycyrrhiza uralensis*, *Citrus reticulata* pericarp, *Citrus aurantium*, *Nelumbo nucifera*, *Cimicifuga foetida* | Leading role | Treat large intestine damp and heat, gestation | Gynecological Treatment of the Zhulin Temple (《竹林女科证治》), 1644–1911 |
| Jia Mi Xiao Ji San | Red yeast rice, *Atractylodes lancea*, *Magnolia officinalis*, *Citrus reticulata* pericarp, *Glycyrrhiza uralensis*, medicated leaven, *Crataegus pinnatifida*, *Hordeurn vulgare* | Leading role | Treat injury of spleen, diarrhea | Zheng Yin Mai Zhi (《症因脉治》), 1706 |
| Ju He San | Red yeast rice, *Citrus reticulata* seed, *Psoralea corylifolia*, *Boswellia carterii*, *Corydalis yanhusuo*, *Artemisia vulgaris*, *Commiphora myrrha* resin, *Acanthopanax gracilistylus* | Leading role | Treat lumbago, smolder | An Outline of Medicine (《医学纲目》), 1565 |
| Qing Liu Wan | Talcum, *Glycyrrhiza uralensis*, red yeast rice | Supporting role | Treat dampness-heat, diarrhea | Danxi’s Experiential Therapy (《丹溪心法》), 1481 |
| Yi Leng Wan | *Arca subcrenata*, calcite, mirabilite, red yeast rice, koji, *Rhizoma pinelliae*, endothelium corneum gigeriae galli, *Corydalis yanhusuo*, pig pancreas | Supporting role | Treat deficiency and stagnation of intestines and stomach | Yi Ji, (《医级》), 1777 |
| Xin Huang Pian | *Sarcandra glabra*, *Panax pseudoginseng*, calculus bovis, pig biliary, *Urena lobate*, pearl powder, cornu bubali, red yeast rice, indometacin | Supporting role | Treat damp-heat, blood stasis, pharyngalgia, toothache, jaundice, costalgia | Chinese Pharmacopoeia, 2015 |
